# Dissecting the genetic and phenotypic basis of salinity tolerance in mungbean: insights from multi-stage phenotyping, GWAS and genomic prediction

**DOI:** 10.1007/s00122-025-04983-z

**Published:** 2025-08-09

**Authors:** Md Shahin Iqbal, Candy M. Taylor, Lukasz Kotula, Al Imran Malik, William Erskine

**Affiliations:** 1 Centre for Plant Genetics and Breeding, The UWA School of Agriculture and Environment, The University of Western Australia, Perth, WA 6009 Australia; 2 The UWA Institute of Agriculture, The University of Western Australia, Perth, WA 6009 Australia; 3 Pulses Research Center, Bangladesh Agricultural Research Institute, Ishurdi, Bangladesh; 4 The Commonwealth Scientific and Industrial Research Organisation (CSIRO), Agriculture and Food, Floreat, WA 6010 Australia; 5 International Center for Tropical Agriculture (CIAT-Asia), Lao People’s Democratic Republic Office, Vientiane, Laos

## Abstract

**Key message:**

Mungbean germplasm collection showed diverse responses to salinity stress at vegetative and reproductive stages. GWAS identified stage-specific genetic associations and candidate genes in the first genetic study of salinity tolerance in mungbean across these stages.

**Abstract:**

Mungbean is an important grain legume widely grown in rice-based farming systems of South and Southeast Asia. Salinity stress severely limits mungbean growth and yield, with cultivars differing widely in susceptibility. This study evaluated phenotypic responses and genetic diversity for salinity tolerance in a mungbean mini-core germplasm collection at early vegetative, late vegetative and reproductive stages, grown in soil-filled pots exposed to control (non-saline) and 75 mM NaCl treatments in a temperature-controlled glasshouse. Salinity stress significantly reduced growth, seed yield and related traits, highlighting distinct phenotypic and genotypic responses across growth stages. Genome-wide association studies and genomic prediction (GP) were performed using two SNP datasets: 5991 DArTseq SNPs and 198,474 Illumina whole-genome resequencing (WGRS) SNPs. A range of 18–22 significant genetic associations were identified in the three growth stages, but none were common across these stages. Both SNP datasets showed distinct genomic regions associated with salinity tolerance traits. GP showed potential to predict salinity tolerance-associated traits. Despite their lower genome-wide density, DArTseq SNPs performed similarly to high-density WGRS SNPs in association analyses and GP accuracy, highlighting their potential as a cost-effective genotyping system for efficient and practical commercial breeding applications. Evaluating the effects of significant SNPs revealed seven functional SNPs linked with seven candidate genes encoding callose synthase, ethylene receptor, dynamin-related protein, cytochrome P450, bHLH-type transcription factor and Kinesin-10-type motor protein. The findings demonstrate need for stage-specific breeding approaches and highlight novel genetic resources (including markers and germplasm) for enhancing salinity tolerance in mungbean.

**Supplementary Information:**

The online version contains supplementary material available at 10.1007/s00122-025-04983-z.

## Introduction

Mungbean [*Vigna radiata* (L.) R. Wilczek var. *radiata*], also known as green gram, is a self-pollinated, warm-season, annual legume crop with a diploid (2x = 22) genome size of 579 Mb (Kang et al. 2014). Mungbean is one of the most nutritionally important grain legumes given it is rich in easily digestible proteins (24–28% on a dry weight basis), dietary fibre (3.5–4.5%), fat (1.0–1.5%), ash (4.5–5.5%), carbohydrates (59–65%) and energy (334–344 kcal), and contains higher levels of folate and iron than most other legumes (Mehandi et al. 2019; Hou et al. 2019). It is widely grown in rice-based cropping systems of South and Southeast Asia for its multiple uses as edible seeds and sprouts, forages, and for soil fertility enhancement. Originating from South Asia, mungbean cultivation has expanded globally to regions like Australia and East Africa (Nair and Schreinemachers 2020) due to its short life cycle (65–90 days), low input requirement and high global demand. The global cultivation area spans about 7.3 million hectares, with an average yield of 721 kg ha^–1^ (Nair and Schreinemachers 2020). However, the crop faces productivity challenges due to its vulnerability to climate change, which increases its sensitivity to biotic and abiotic stressors like soil salinity.

Salinity severely threatens crop production, especially in arid and semi-arid regions (Shahid et al. 2018), and currently affects around 20% of cultivated land, with projections indicating a further rise in affected land by the mid-twenty-first century (Mukhopadhyay et al. 2021). Excessive soluble salts and exchangeable sodium ions in the soil rhizosphere zone induce osmotic stress and ionic toxicity and restrict nutrient uptake (Munns 2002). Mungbean growth and yield are severely impacted by salinity stress, with a reduction of 41–91% in yields when grown in 50 mM NaCl treated pot soil in glasshouse (Sehrawat et al. 2015). Salinity adversely affects mungbean throughout all stages of its life cycle, including germination (Breria et al. 2020b), vegetative growth (Alharby et al. 2019) and, particularly, reproductive processes (Sehrawat et al. 2014a; Manasa et al. 2017). Specifically, salinity stress causes chlorosis and necrosis symptoms, and reduces root and shoot lengths, leaf number and area, flower and pod numbers, seed number per pod, 100-seed weight and seed yield (Manasa et al. 2017; Alharby et al. 2019; Liu et al. 2022; Iqbal et al. 2024b). While varietal responses to salinity stress have been studied extensively during germination and early seedling stages (Sehrawat et al. 2014b; Breria et al. 2020b; Liu et al. 2022), exploration of responses during later vegetative growth stages and the reproductive stage has been limited. A study on mungbean responses to salinity (150 and 300 mM NaCl) grown in pot soil in a glasshouse found significant genotypic variation in biomass and pod yield (Manasa et al. 2017). However, the small sample size in this study (40 breeding lines) and the crop’s high sensitivity to salinity stress resulted in a limited range of genotypic variation for mungbean salinity tolerance being observed, emphasising the need for systematic screening of diverse germplasm to elucidate genetic variation in salinity tolerance within the crop species.

Growth and yield reductions due to excessive soil salinity result from osmotic stress and ion toxicity (Munns 1993; Munns and Tester 2008; Roy et al. 2014). The osmotic stress starts immediately upon exposure to increased salt concentrations in the root zone. This stress limits water uptake, reduces cell turgor and lowers stomatal conductance, which inhibits leaf and shoot elongation. Consequently, there is a reduction in leaf area and overall shoot growth (James et al. 2008; Munns and Tester 2008; Rahnama et al. 2010; Roy et al. 2014). The osmotic effects of salinity stress affected growth responses in durum wheat (*Triticum turgidum* L. ssp. *durum* Desf.; Rahnama et al. 2010) and barley (*Hordeum vulgare* L.; Saade et al. 2020) at 10 and 11 days after salt imposition, respectively. When dissolved salts in the soil solution enter the plant and accumulate in plant tissues reaching high concentrations over time (several days to weeks), ion toxicity can occur (Munns 1993). High shoot Na^+^ and Cl^–^ concentrations inhibit photosynthesis in mungbean, reducing vegetative growth and yield (Wahid et al. 2004; Syeed and Fatma 2011; Le et al. 2021; Iqbal et al. 2024a, b). The ionic component of salinity stress significantly affected growth in durum wheat (James et al. 2002) and mungbean (Iqbal et al. 2024a, b) at 27 and 23 days of salt treatment, respectively. However, genotypic variation and the effects of osmotic stress and ion toxicity on mungbean growth and development are yet to be fully explored.

Salinity tolerance is a complex, genetically controlled quantitative trait influenced by multiple genes (Roy et al. 2014; van Zelm et al. 2020). Identifying quantitative trait loci (QTLs) associated with salinity tolerance aids in developing cultivars with enhanced tolerance. While QTL discovery for salinity tolerance has been reported in various legumes, including chickpea (*Cicer arietinum* L.), soybean (*Glycine max* L.), cowpea (*Vigna unguiculata* (L.) Walp. and field pea (*Pisum sativum* L.) (reviewed in Jha et al. 2019), limited information is available for mungbean. Genome-wide association studies (GWAS) represent a powerful strategy to identify genomic regions controlling complex quantitative traits and detect alleles and associated markers for exploitation in varietal improvement. Compared with conventional QTL mapping, GWAS relies on historical linkage disequilibrium (LD) between marker–trait associations across all chromosomes in a natural population of diverse germplasm (Korte and Farlow 2013). It also has superior resolution to enable large-scale and high-precision identification of elite alleles and allelic variations distributed in natural populations (Zhao et al. 2011).

Over the past decade, GWAS has been used extensively in many legume species to identify genetic factors governing complex traits, such as salinity tolerance in mungbean at the seedling stage (Breria et al. 2020b; Liu et al. 2022), soybean (Do et al. 2019), cowpea (Ravelombola et al. 2018) and *Medicago truncatula* (Kang et al. 2019). Breria et al. (2020b) identified two genomic regions on chromosomes 7 and 9 associated with seed germination in mungbean under salinity stress (50 mM NaCl). Recently, Liu et al. (2022) identified significant marker–trait associations distributed on chromosomes 1, 3 and 5 for survival rate in mungbean 10 and 15 days after a 100 mM NaCl treatment. However, no genetic studies have explored salinity tolerance in mungbean during the vegetative and reproductive stages. Salinity tolerance at one growth stage may not translate to other stages in mungbean, as salinity responses vary depending on genotype, growth stage and salt concentration (Iqbal et al. 2024b).

Despite the power of GWAS to detect significant associations, the effects of rare variants on phenotypic variation of complex traits cannot be detected and remain recalcitrant to conventional marker-assisted selection (Lipka et al. 2015). Genomic prediction (GP) can capture the effect of rare variants, as GP models include all genome-wide markers rather than a few large-effect markers, as in the marker-assisted selection model (Meuwissen et al. 2001). GP has great potential to accelerate genetic gain by shortening the breeding cycle for traits that are difficult to phenotype or possess complex genetic architectures, such as yield under salinity stress. Various approaches have been explored to develop GP models, including comparisons of statistical methods, marker densities, markers from different genotyping platforms, and the integration of GWAS-based markers to enhance predictive accuracy (Alemu et al. 2024; Crossa et al. 2024). Despite the successful application of GP in various crop species to improve adaptive traits, such as salinity tolerance in wheat (*Triticum aestivum* L.; Javid et al. 2022), drought tolerance in chickpea (Li et al. 2018), and agronomic (Beche et al. 2021) and yield-related (Bhat et al. 2022) traits in soybean, its use in mungbean remains unexplored.

This study leverages a mungbean mini-core germplasm collection representing the maximum diversity of the 6700 mungbean accessions at the World Vegetable Center (Schafleitner et al. 2015). This mini-core collection has been genotyped using a reduced-representation genotyping-by-sequencing (GBS) approach known as Diversity Arrays Technology (DArT) (Breria et al. 2020a) and successfully used for GWAS for several traits in mungbean, including seed coat luster (Breria et al. 2020a), hypocotyl colour (Sokolkova et al. 2020), 100-seed weight (Akhtar et al. 2021), root traits (Aski et al. 2021), salinity tolerance at the germination stage (Breria et al. 2020b), and waterlogging tolerance at the germination and seedling stages (Kyu et al. 2024). Recently, the mini-core collection was re-genotyped by the World Vegetable Center using whole-genome resequencing (WGRS) to provide high-density genome-wide marker coverage. This study evaluated phenotypic responses and genetic variation in the mungbean mini-core collection for salinity tolerance during three unexplored growth stages: early vegetative, late vegetative and reproductive stages. To distinguish between responses to the osmotic and ionic components of salinity stress, we investigated the effects of salinity on shoot growth 15 days after salinity treatment (early vegetative stage) and 30 days after salinity treatment (late vegetative stage). We also investigated salinity stress responses up to maturity to study yield-related traits. The SNP markers derived from DArTseq and WGRS approaches were used to compare QTL discovery and GP accuracy for salinity tolerance in the mungbean mini-core collection. We also investigated the potential of using GWAS-based markers in GP models to assess their practical utility in genomic selection. The study aimed to (i) assess phenotypic responses and genetic variation for salinity tolerance in mungbean during vegetative and reproductive stages, (ii) identify genomic region(s) and candidate gene(s) associated with salinity tolerance in mungbean at the vegetative and reproductive stages and (iii) investigate whether high-density markers in GWAS improve the precision of association mapping and whether high-density markers or incorporating GWAS-based markers into GP enhance predictive accuracy for salinity tolerance in mungbean.

## Materials and methods

The study comprised two sets of experiments to evaluate the phenotypic responses for salinity tolerance in mungbean at the early vegetative, late vegetative and reproductive stages.

### Experiment A: evaluation of salinity tolerance at the late vegetative stage

A panel of 292 mini-core genotypes from the World Vegetable Center (Schafleitner et al. 2015) were used to assess phenotypic responses for salinity tolerance in mungbean at the late vegetative stage. Seeds were obtained from the Department of Agriculture and Fisheries, Queensland. The geographical origin of these genotypes spans ten regions with most originating from South Asia (209), South West Asia (34) and South East Asia (20) (Supplementary Tables S1 and S2).

**Fig. 1:**
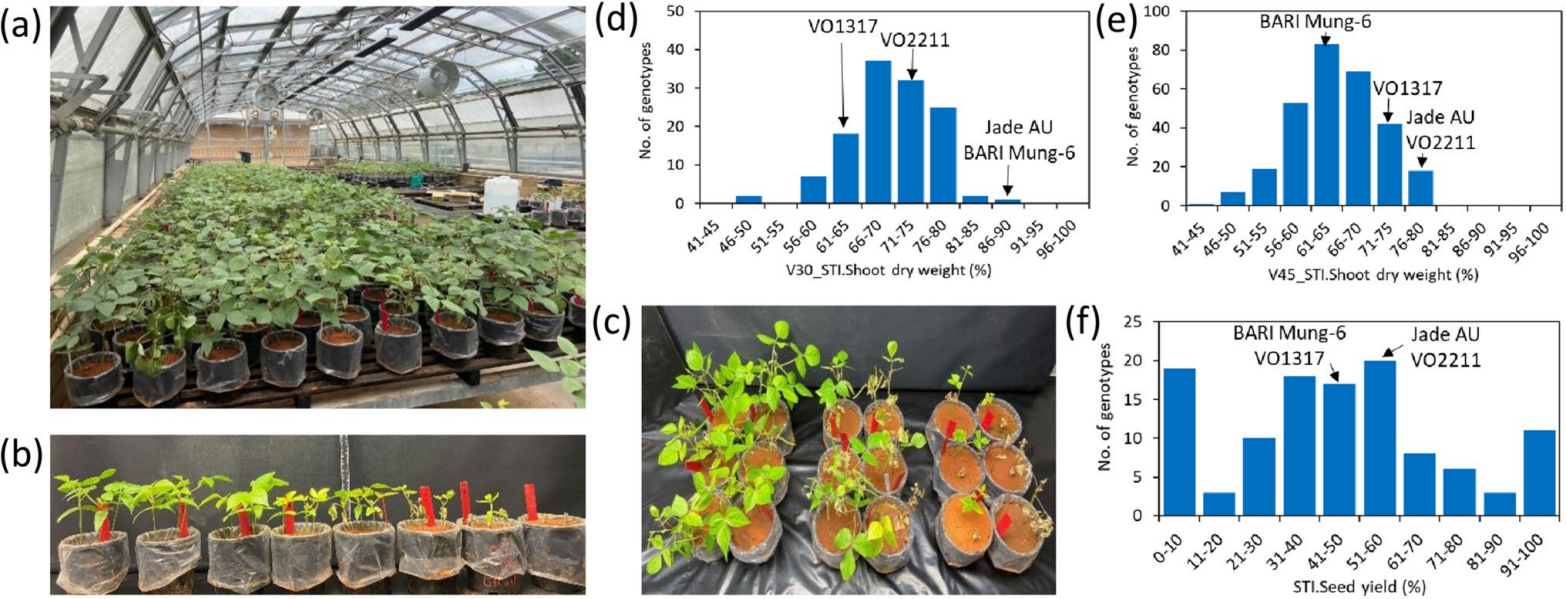
Phenotyping of mungbean mini-core genotypes for salinity tolerance (**a**). Plants were grown in soil with 0 (non-saline control) and 75 mM NaCl treatments imposed on 15 DAS. Contrasting mungbean genotypes grown in 75 mM NaCl at early vegetative stage (30 DAS; **b**) and reproductive stage (**c**). Frequency distribution for the salinity tolerance index (STI, % of control) of V30_STI.Shoot dry weight at early vegetative stage (30 DAS; **d**), V45_STI.Shoot dry weight at late vegetative stage (45 DAS; **e**) and STI. Seed yield (**f**) of mungbean mini-core genotypes. Arrows indicate the salinity tolerance index values for the check varieties

The experiment was conducted between March and August 2021 in a controlled temperature glasshouse (Fig. 1a) at The University of Western Australia, Perth, WA, Australia (31°57’S, 115°47’E). During this period, the diurnal temperature range was 30 ± 3/24 ± 2 °C day/night, with 10–12 h day length and a maximum PAR of 1400–1650 μmol m^−2^ s^−1^. Plants were grown in free-draining plastic pots (180 mm diameter, 180 mm high) lined with waterproof polybags and filled with 3.15 kg of 2 mm-sieved oven-dried red-brown sandy clay loam soil (pH = 8.79, electrical conductivity 0.28 dS m^–1^ in a 1:5 soil/water extract) collected from Mukinbudin, Western Australia (Iqbal 2024). The water content (w/w) at field capacity was 19.7%. The soil was fertilised as per Iqbal (2024). Nutrients were added to the soil as (g pot^–1^): 0.41 K_2_SO_4_, 0.71 CaSO_4_.2H_2_O, 0.60 KH_2_PO_4_, 0.08 MgSO_4_.7H_2_O, and 2.21 mL of half-strength Hoagland solution micronutrients with deionised distilled water in a sufficient solution volume to wet the soil to 80% field capacity before sowing. Seeds were surface sterilised with 1% commercial bleach (active ingredients NaOCl, 40 mg L^–1^) for 1 min, rinsed with deionised water for four minutes and then pre-germinated on filter paper moistened with deionised water in Petri dishes for 12 h in the dark. For each pot, six seeds were sown at 30 mm depth along with peat-based Group I mungbean *Rhizobium* (*Bradyrhizobium* spp.) strain CB 1015 (5.25 g pot^–1^; Group N, New Edge Microbials Pty Ltd, Albury, New South Wales, Australia). Pots were watered with deionised water to maintain 80% field capacity every alternate day. Seedlings were thinned to three per pot seven days after sowing (DAS).

Two treatments were used: 0 (non-saline control) and 75 mM NaCl. The 75 mM NaCl treatment was used based on a pilot study where 75 mM NaCl was sufficient to discriminate between sensitive and tolerant genotypes (Iqbal et al. 2024a, b). Moreover, the 75 mM NaCl treatment represents moderate salinity stress, equivalent to approximately 7.5 dS/m, which is commonly encountered in agricultural soils of semi-arid and irrigated regions affected by salinisation (Iqbal 2024). The experiment had an alpha-lattice design with two factors (292 genotypes × 2 treatments) and three biological replications. A spatial row-column blocking design was used to reduce the positional effect in the glasshouse within replicates and improve the precision of treatment comparisons. The experiment was repeated three times due to space limitations for phenotyping. Each repetition involved 292 genotypes, with each considered one biological replication. Within each replication, different benches in the glasshouse were considered as separate blocks in alpha-lattice design where individual pots were arranged in 10 rows and 6 columns. The three repetitions had uniform growing conditions, including soil and experimental procedures. The three repetitions were planted in the first week of March, May and July 2021, respectively. The salinity treatments were imposed 15 DAS when most plants were approximately at the V1 stage (i.e. unifoliate leaves attached to the first node are fully expanded and flat as the first trifoliate leaf attached to the upper node starts to unroll; Pookpakdi et al. 1992). The NaCl treatment was stepped up by the addition of 25 mM NaCl (0.288 g NaCl kg^–1^ soil) progressively over three days to reach 75 mM NaCl. NaCl was applied to pots in a sufficient solution volume to wet the soil to 80% field capacity, with the equivalent volume of deionised water added to non-saline control pots. The pots were weighed every alternate day and watered with deionised water to 80% field capacity for the experimental duration. The salinity treatment was maintained for 30 days after the first treatment application (15 DAS), and plants were harvested at 45 DAS when most genotypes were at the late vegetative stage and some were at the early reproductive stage.

At the time of harvest, foliar injury was assessed using a visual scale adapted from Maliro et al. (2008), where:

1. No injury symptoms
2. Leaf with slight chlorosis or beginning to yellow
3. Chlorosis or overall yellowing on 25% of plant
4. Necrosis beginning on 25% of plant
5. Necrosis on 25% of plant or chlorosis on 50% of plant
6. Necrosis more than 50% of plant or chlorosis on 75% of plant
7. Necrosis more than 75% of plant or chlorosis on whole plant
8. Necrosis on whole plant and very youngest leaves still green
9. Only stem and shoot tips green
10. Plant dead, no green parts

Leaf chlorophyll concentration in leaflets of the youngest fully expanded leaves (YFEL) was measured using a SPAD meter (Minolta, Osaka, Japan) at 45 DAS. Each plant’s final chlorophyll meter reading was the average of three technical readings. Plant height (cm) from the base of the stem to the apical meristem of all plants was recorded at 45 DAS. One YFEL (three leaflets) was harvested from three plants and weighed. Then, plants were cut at the soil surface level to record shoot fresh weight before oven-drying all samples at 65 °C for 72 h to record dry weight. Data were collected on a per-plant basis. Leaf water content was calculated as: fresh mass of YFELs—dry mass of YFELs and expressed as mL g^–1^ dry mass.

### Experiment B: evaluation of salinity tolerance at the early vegetative and reproductive stages

A subset of 124 mini-core genotypes was selected based on the phenotyping results of the screening experiment at the late vegetative stage (i.e. Experiment A) to evaluate salinity tolerance at the early vegetative and reproductive stages. From Experiment A, 292 genotypes were grouped into five distinct tolerance groups based on shoot dry weight reduction compared to non-saline control at the late vegetative stage. Subsequently, 20–30 genotypes were selected from different geographical regions within each tolerance group to ensure maximum diversity among genotypes in Experiment B. The geographical origin of these 124 genotypes was presented in Supplementary Tables S1, S3 and S4.

The experiment was conducted between mid-October 2021 and January 2022 in the same controlled glasshouse with similar growth conditions and experimental procedures as Experiment A except for a 14-h day length. Two treatments were used: 0 (non-saline control) and 75 mM NaCl. The experiment had an alpha-lattice design with two factors (124 genotypes × 2 treatments) and three biological replications. The spatial row-column blocking design was used to reduce the positional effects in the glasshouse within replicates. The salinity treatments were imposed 15 DAS and maintained until harvest.

Two harvests were made: first harvest at 30 DAS (after 15 days of salinity treatment imposed when all the genotypes were at the early vegetative stage) and second harvest at maturity. At the first harvest, foliar leaf injury, leaf chlorophyll concentration and plant height were recorded on three plants using the same method as Experiment A. One randomly selected plant from each pot and its YFEL were harvested separately and subsequently photographed with a digital camera to measure leaf size area (cm^2^) using ImageJ software. Shoots were oven-dried at 65 °C for 72 h to record dry weight. The remaining two plants were grown to maturity. Time to 50% flowering and 50% pod maturity were recorded for each plant. Due to the asynchronisation of maturity in mungbean, mature pods were harvested twice and combined. Pod number/plant, mature seeds/pod and pod length were recorded. All mature seeds/plant were pooled and weighed, with 100-seed weight calculated as the weight of mature seeds/the number of mature seeds. Aboveground samples, including pod walls and immature seeds, were weighed and included in shoot mass measurements.

### Phenotypic data analysis

Phenotypic traits were analysed using a linear mixed model (LMM) with residual maximum likelihood (REML) estimation that appropriately partitioned and accounted for genetic and non-genetic sources of variation (Gilmour et al. 1997) in ASReml-R V4.1.0.176 (Butler et al. 2018). An LMM for each trait was fitted by adding genotype and genotype × salinity treatment interaction as random effects and salinity treatment as the fixed effect. Replications and spatial components (rows and columns) were added as random terms. Residual plots, sample variograms, row and column faces of the empirical variogram, and log-likelihood ratio tests were used to check model accuracy and select the best model to describe the data. By partitioning the additive genetic variance (V_A_) and residual variance (V_R_), V_A_ and V_R_ were estimated for control and salinity treatment. Broad-sense heritability (H^2^) was calculated as follows:

$${\text{H}}^{2}= \frac{{\text{V}}_{\text{A}}\text{ for control or salinity}}{{\text{V}}_{\text{A}}\text{ for control or salinity}+{\text{V}}_{\text{row}} + {\text{V}}_{\text{column}} + \frac{{\text{V}}_{\text{R}}\text{ for control or salinity}}{\text{number of replications}}}$$

From the fitted LMM, best linear unbiased predictors (BLUPs) of the individual genotype for the control and salinity treatments for all traits were extracted and used to perform further tests. The salinity tolerance index (relative value, RV of salinity tolerance) was calculated using BLUPs of all measured traits using the formula:

$$\text{Salinity tolerance index }\left(\text{STI}\right)= \frac{\text{BLUPs under salinity stress}}{\text{BLUPs under control}}\times 100$$

An alternative LMM was fitted to test the significance of the salinity tolerance index for all traits. Firstly, BLUPs were calculated from each replication using the same model as above, and salinity tolerance indices were calculated. Then, LMM was fitted with the salinity tolerance index value by adding genotype as the fixed effect and error as the random effect. The significance was tested using the appropriate Wald statistics. Repeated measures ANOVA was used to test for differences in the salinity index for plant height and shoot dry weight between the early vegetative and reproductive growth stages. Descriptive statistics were analysed using ‘pastecs’ package V1.3.21 (Grosjean and Ibanez 2018), and a correlation plot was created using ‘corrplot’ package V0.93 (Wei and Simko 2017) in R 4.2.2 (R Core Team 2022). A one-way ANOVA was also performed by geographical region of origin.

### Genotyping and linkage disequilibrium (LD)

Two sources of genotypic markers—DArTseq- and WGRS-derived SNPs—were used for the genetic analysis of the 292 mungbean mini-core genotypes. A total of 200,321 raw SNPs were generated using WGRS at the World Vegetable Center. The raw WGRS sequence reads were aligned to the new mungbean reference genome, Crystal (unpublished data accessed from World Vegetable Center: https://avrdc.org/dna-sequence-data-download) to establish the physical positions of the SNPs. The SNP markers were filtered based on the minor allele frequency < 5%, maximum allele heterozygosity of 20%, and call rate < 10% using Trait Analysis by Association, Evolution, and Linkage (TASSEL) V5.2.87 (Bradbury et al. 2007). Finally, 198,474 SNPs were retained and used for further analysis. A second SNP dataset of 24,870 raw DArTseq SNP markers was obtained from the World Vegetable Center (Breria et al. 2020a). The raw sequence tags were similarly aligned to the new mungbean reference genome, Crystal, to establish physical positions for SNPs using the gl.blast function in ‘dartR’ package V2.7.2 (Mijangos et al. 2022) in R 4.2.2, with 5,991 high-quality SNPs selected based on filtering (minor allele frequency of 5% and call rate of 50%) in TASSEL software. For both marker datasets, pairwise LD among the markers was calculated as a squared allele frequency correlation (*r*^2^) between SNP marker pairs in TASSEL with a sliding window of 50 markers. The extent of genome-wide LD decay was examined by plotting average *r*^2^ values against the physical position of the SNPs in R 4.2.2. A locally weighted polynomial regression (LOWESS) curve was drawn to display LD decay, with the LD decay distance determined when the average pairwise *r*^2^ declined to half of its maximum value.

### Population structure and genetic diversity analysis

Population structure and genetic diversity were explored separately using the two marker datasets. Principal component analysis (PCA) was performed using Genomic Association and Prediction Integrated Tool (GAPIT) version 3 (Lipka et al. 2012), and the PCA graph was plotted using the ggplot2 package in R 4.2.2. The optimum number of principal components (PCs) that adequately explained population structure was determined by the elbow point of the scree plot generated by GAPIT (Cattell 1966). Population structure was analysed using filtered SNPs in the ‘LEA’ package in R 4.2.2, similar to the Bayesian clustering program of STRUCTURE (Frichot and François 2015). Shared ancestry among individuals were examined using models of K ranging from 1 to 10, with each model repeated three times. Population structure was plotted using the ‘barplot’ function in R 4.2.2. Genotypes with family relation coefficients (*Q* value) > 70% were considered a subgroup, and those with < 70% were defined as being an admixture (Breria et al. 2020a). A neighbour-joining dendrogram was constructed using only the WGRS-derived SNPs based on the genetic distance kinship matrix from GAPIT output and visualised in Iroki (Moore et al. 2020). Information on the geographical origin of the 292 mini-core genotypes was added as a colour code in the dendrogram. Nei’s genetic diversity and polymorphism information content (PIC) values for each marker were calculated as described in Nagy et al. (2012). Briefly, Nei’s genetic diversity (H) = 1 – P^2^ – (1 – P)^2^ and PIC = H – 2 × P^2^ (1 – P)^2^, where P is the frequency of one of the two alleles, and because the SNP is bi-allelic, (1 – P) is the frequency of the other allele.

### Genome-wide association study (GWAS)

GWAS was independently conducted using both sources of filtered SNP markers (i.e. DArTseq and WGRS) and the salinity tolerance index of 21 phenotypic traits in GAPIT version 3 (Lipka et al. 2012). The association analysis was tested by fitting five different statistical models, including general linear model (GLM; Price et al. 2006), mixed linear model (MLM; Yu et al. 2006), compression MLM (CMLM; Zhang et al. 2010), fixed and random model circulating probability unification (FarmCPU; Liu et al. 2016), and Bayesian-information and Linkage disequilibrium Iteratively Nested Keyway (BLINK; Huang et al. 2019). The most appropriate model was selected based on inspection of Q-Q plots for a lack of P-value inflation. Finally, GWAS was conducted using the BLINK model (determined as the most appropriate) with the population structures: Q1 and Q2 for DArTseq-derived SNPs and Q1, Q2 and Q3 for WGRS-derived SNPs as covariates for all the traits. A GWAS threshold P-value was determined based on the equation *α* = 1/m, where m is the total number of markers for 5,991 DArTseq SNPs [− log_10_ (*P*-value = 1.89 × 10^−4^) ≤ 3.7] and for 198,474 WGRS SNPs [− log_10_ (*P*-value = 5.03 × 10^−6^) ≤ 5.2] to declare significant marker–trait associations. The false discovery rate (FDR threshold) was also determined based on the proportion of false positive and false negative associations. Manhattan plots were visualised using the ‘qqman’ package (Turner 2014) in R 4.2.2. Linkage disequilibrium blocks were identified for each significantly associated SNP and visualised using Haploview 4.1 software (Barrett et al. 2005) using a confidence interval method with *r*^2^ < 0.8. Predictions of SNP effects were performed using The Ensembl Variant Effect Predictor (McLaren et al. 2016) using the Crystal reference genome and gene annotation GFF3 file (accessed from the World Vegetable Center: https://avrdc.org/dna-sequence-data-download).

### Identification of candidate genes

Significantly associated SNPs and their respective candidate genes were viewed in the mungbean reference genome assembly for variety Crystal using the Integrative Genomics Viewer V2.15.4 (Robinson et al. 2011). All annotated genes located within 193 Kb (i.e. the more stringent LD decay distance established in this study using the WGRS dataset) upstream and downstream of each significant SNP were identified as positional candidate genes. Significant SNPs were further examined to see if they cause a non-synonymous mutation within the coding sequence of the positional candidate genes to determine whether they may also be of interest from a functional perspective. Arabidopsis and soybean homologs for each candidate gene were extracted using ‘blastn’. Homologous sequences were submitted to the ‘agriGO v2.0’ gene ontology enrichment facility (http://systemsbiology.cau.edu.cn/agriGOv2/species.php). Gene ontology (GO) enrichment analysis was performed based on a Fisher’s exact test and a Yekutieli multitest adjustment of 5% false positive detection threshold to identify GO terms that were significantly most represented among the salinity stress-regulated genes compared to the Crystal reference genome.

### Genomic prediction

The ridge regression best linear unbiased prediction (RR-BLUP) model (Meuwissen et al. 2001) was used to conduct GP as a base model to compare prediction accuracies fitted with different set of SNPs. Although GP was explored for all three growth stages examined in this study, the smaller sample sizes in the early vegetative and reproductive stages (124 genotypes each) created unacceptably large standard errors. Therefore, only analyses for the six phenotypic traits are presented at the late vegetative stage, where a larger sample size (292 mini-core genotypes) was used and lower standard error were encountered. Initially, we evaluated models fitted with either all DArTseq (5991 SNPs) or all WGRS SNPs (198,474 SNPs). Secondly, a pruned subset of WGRS SNPs (5917 SNPs) selected on marker quality (PIC > 0.26) were used to test the prediction accuracies whether the high dimensionality of using all WGRS SNPs reduced the statistical power of accurately estimating marker effects. Thirdly, we combined the all DArTseq SNPs with pruned WGRS SNPs to test the potential of integrating data from different sequencing platform. Lastly, we investigated the possibility of integrating GWAS results into GP model. Based on the GWAS results of the six phenotypic traits at the late vegetative stage, SNPs with lowest P values (less than 0.05 and 0.005) were selected from both DArTseq and WGRS SNPs based GWAS results and combined.

A fivefold cross-validation was performed to evaluate the prediction performances. The 292 mini-core genotypes were randomly divided into five subsets (i.e. folds), each with equal number of genotypes. All of the GP and GWAS models previously described were fitted in the four of five folds (training set, 90%), and then, the predictive ability was tested in the fifth fold (validation set, 10%). This schemes was repeated five times. Predictive accuracies were calculated as Pearson’s correlation coefficient between the predicted genomic estimated breeding values (GEBVs) and true phenotypic values of the validation set and an average predictive accuracy was reported. The GP models was implemented in the R package ‘rrBLUP’ (Endelman 2011).

## Results

### Phenotypic variation of salinity tolerance

#### Early vegetative stage

**Fig. 2:**
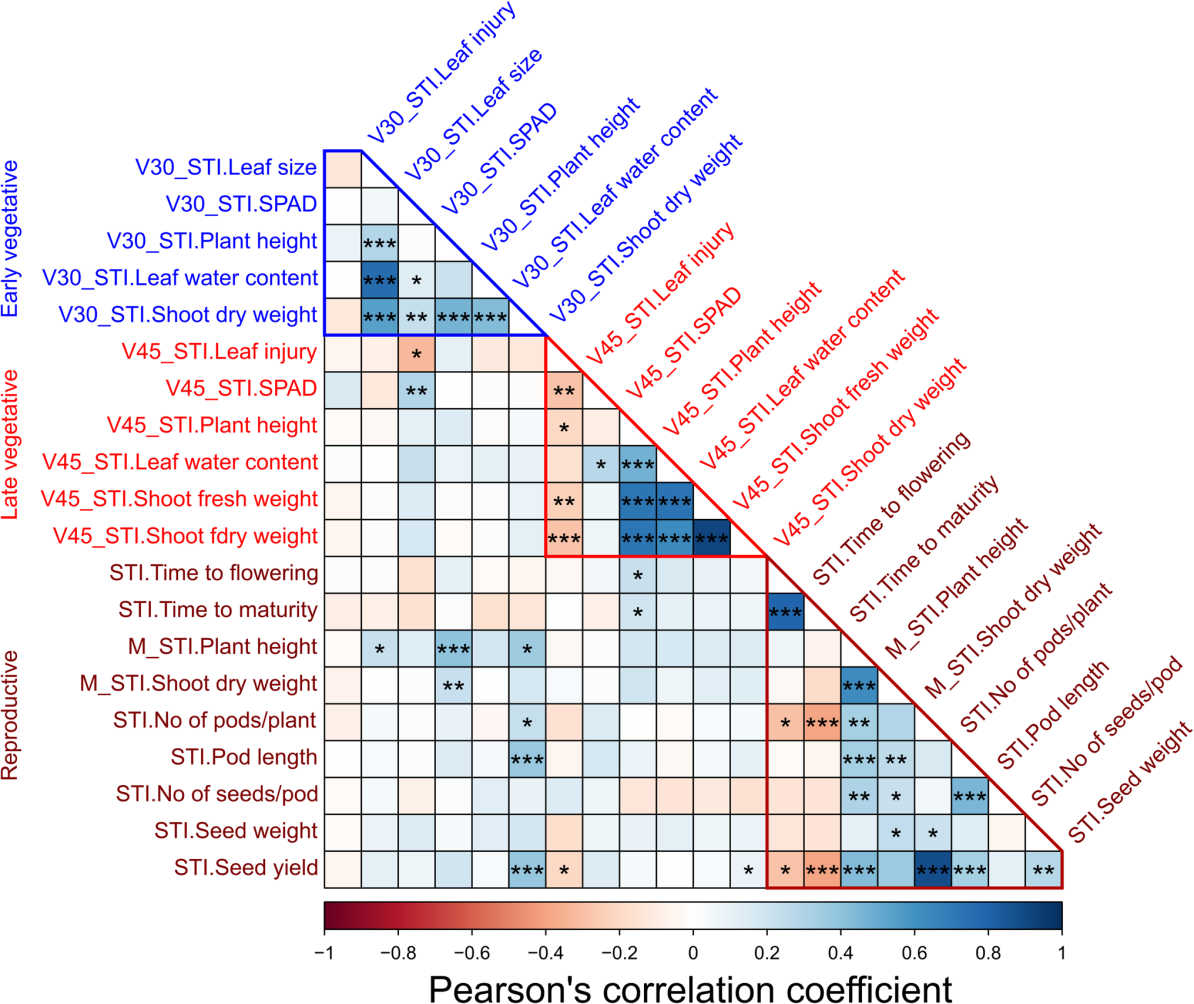
Pearson’s phenotypic correlation coefficients among salinity tolerance index of various traits at the early vegetative (30 DAS), late vegetative (45 DAS) and reproductive stages. Colour scale represents the value of the correlation coefficient. *, **, *** indicate significance at *P* < 0.05, *P* < 0.01 and *P* < 0.001, respectively

REML analysis showed significant effects of the salinity treatments, genotypes and genotype × treatment interaction for all traits at the early vegetative stage (Supplementary Table S5). The estimates of variance components showed that salinity treatment accounted for the highest proportion of observed variation for all traits. Overall, the salinity treatment increased leaf injury symptoms and leaf water content and reduced other growth-related traits (Fig. 1b), with shoot dry weight more affected (70% of controls) than leaf size (93% of controls), SPAD (88% of controls) and plant height (88% of controls) (Table 1). The mungbean genotypes exhibited high variation for all traits under control and salinity treatments (Supplementary Table S6). Low to medium broad-sense heritability (H^2^) was observed, ranging from 18% (SPAD) to 46% (plant height) in the salinity treatment. The ANOVA revealed significant differences among the 124 genotypes at the early vegetative stage for the salinity tolerance index of V30_STI.SPAD, V30_STI.Plant height and V30_STI.Shoot dry weight (Table 1). Different traits showed a broad range of variation, with the greatest variation for V30_STI.Shoot dry weight (48–90%). Frequency distribution of V30_STI.Shoot dry weight showed a continuous distribution (Fig. 1d). Among the 124 genotypes, the salinity treatment decreased shoot dry weight to 81–90% of the controls in three genotypes and 76–80% in 25 genotypes. According to Pearson’s correlation analysis, V30_STI.Shoot dry weight significantly correlated with all traits except V30_STI.Leaf injury (Fig. 2). The ANOVA based on geographical origin showed no significant differences among the regions for all traits at the early vegetative stage (Supplementary Table S7).

**Table 1:** Summary statistics for the salinity tolerance index (STI, % of control) of various traits at the early vegetative stage (30 DAS), late vegetative stage (45 DAS) and reproductive stage

Traits	Mean	Minimum	Maximum	SD	*F*-value
**Early vegetative stage (30 DAS)**					
V30_STI.Leaf injury	236.0	161.4	326.7	31.62	1.00
V30_STI.Leaf size	93.2	66.7	115.0	9.18	0.82
V30_STI.SPAD	88.2	84.9	90.2	0.97	1.02*
V30_STI.Plant height	88.0	76.4	100.0	3.61	0.97*
V30_STI.Shoot dry weight	70.1	48.0	89.7	6.45	1.09*
V30_STI.Leaf water content	124.5	87.0	170.3	13.87	1.04
**Late vegetative stage (45 DAS)**					
V45_STI.Leaf injury	258.6	214.7	316.8	18.58	1.25**
V45_STI.SPAD	89.2	82.4	95.7	2.21	1.20**
V45_STI.Plant height	84.0	71.0	94.1	4.13	1.27***
V45_STI.Shoot fresh weight	72.8	51.4	93.6	7.99	1.32***
V45_STI.Shoot dry weight	64.6	44.8	79.9	7.01	1.21**
V45_STI.Leaf water content	113.7	90.8	141.7	8.95	1.09
**Reproductive stage**					
STI.Time to flowering	108.8	96.3	108.5	2.39	1.04
STI.Time to maturity	107.7	100.5	106.5	1.07	1.03
M_STI.Plant height	72.3	51.1	87.3	5.51	1.28*
M_STI.Shoot dry weight	79.5	69.4	91.2	4.29	1.22*
STI.No of pods/plant	45.5	0.0	100.0	32.03	1.31*
STI.Pod length	98.7	98.3	99.0	0.13	0.95
STI.No of seeds/pod	95.7	93.8	96.6	0.42	0.91
STI.Seed yield	45.4	0.0	100.0	30.33	1.22*
STI.Seed weight	97.8	90.1	107.4	3.63	1.05

^*^, **, *** indicate significance at *P* < 0.05, *P* < 0.01 and *P* < 0.001, respectively

#### Late vegetative stage

At the late vegetative stage, significant effects of the salinity treatments, genotypes and genotype × treatment interactions were observed for all traits except leaf injury (Supplementary Table S5). The estimates of variance components showed that the salinity treatment accounted for the highest proportion of observed variation for all traits. Similar to the early vegetative stage, salinity treatment overall increased leaf injury symptoms and leaf water content and reduced growth-associated traits, with shoot dry weight more affected (65% of controls) than SPAD (89% of controls), plant height (84% of controls) and shoot fresh weight (73% of controls) (Table 1). Mungbean genotypes exhibited high variation for all traits under control and salinity treatments (Supplementary Table S6). Low to medium broad-sense heritability (H^2^) was observed, ranging from 16% (leaf injury) to 57% (plant height) in the salinity treatment. The ANOVA for salinity tolerance index of most traits at the late vegetative stage revealed significant differences among the 292 genotypes, exception for V45_STI.Leaf water content (Table 1). A wide range of variation occurred for the different traits, for example, V45_STI.Shoot dry weight ranged from 45 to 86% with a continuous frequency distribution (Fig. 1e). Among the 292 genotypes, the salinity treatment decreased shoot dry weight to 76–80% of the controls in eighteen genotypes. Correlation analysis showed that V45_STI.Shoot dry weight significantly correlated with all traits except V45_STI.SPAD (Fig. 2). Significant differences among regions of geographical origin occurred for V45_STI.Shoot dry weight (Supplementary Table S7). However, the ANOVAs by regions were highly unbalanced due to under-represented sample numbers from several regions for this growth stage and all others (Supplementary Table S1). Nonetheless, genotypes from South East Asia had the highest meanV45_STI.Shoot dry weight (72%) (Supplementary Fig. S1a).

#### Reproductive stage

REML analysis revealed significant genotype effects for all traits (Supplementary Table S5). Significant salinity treatment effects occurred for most traits, except pod length and 100-seed weight. Significant genotype × treatment interactions were observed for time to 50% pod maturity, pod number/plant and seed yield. The variance component estimates showed that the salinity treatment accounted for the highest proportion of observed variation for most traits, except 100-seed weight, where genotypes accounted for the highest proportion (Supplementary Table S5). Overall, the salinity treatment delayed flowering and maturity by 4–5 days (Supplementary Table S6), severely reduced pod number/plant and seed yield (~ 46% of controls) but did not affect pod length, seed number/pod or seed weight (~ 97% of controls) (Table 1 and Fig. 1c). The salinity treatment also reduced plant height (72% of controls) and shoot dry weight (80%) at maturity (Table 1). Mungbean genotypes exhibited high variation for all traits in the control and salinity treatments (Supplementary Table S6). Medium to high broad-sense heritability (H^2^) ranged from 34% (plant height) to 83% (100-seed weight) in the salinity treatment. The ANOVA revealed significant differences among the 124 genotypes for the salinity tolerance index of M_STI.Plant height, M_STI.Shoot dry weight, STI.No of pods/plant and STI.Seed yield (Table 1). Large variation was observed for M_STI.Plant height (51–87%), M_STI.Shoot dry weight (69–91%), STI.No of pods/plant (0–100%) and STI.Seed yield (0–100%) (Table 1). STI.Seed yield displayed a scattered frequency distribution (Fig. 1f). Among the 124 genotypes, eleven highly tolerant genotypes had a high STI.Seed yield (91–100%) while nineteen highly sensitive genotypes failed to produce seeds in the salinity treatment (Fig. 1f). Pearson’s correlation analysis revealed that STI.Seed yield was significantly correlated with most traits except M_STI.Shoot dry weight and STI.No of seeds/pod (Fig. 2). Significant differences among the regions of geographical origin occurred for STI.Pod length and STI.Seed yield (Supplementary Table S7). Genotypes from South East Asia had the highest STI.Seed yield (78%), while genotypes from North America had the lowest (20%) (Supplementary Fig. S1b). However, genetic variation among the other regions was negligible, as two-thirds of the genotypes came from South Asia.

#### Relationship of salinity tolerance among the growth stages

Phenotypic correlation analysis showed that V30_STI.SPAD at the early vegetative stage significantly correlated with V45_STI.SPAD (*r* = 0.23**) and V45_STI.Leaf injury (*r* = –0.25*) at the late vegetative stage. STI.Seed yield significantly correlated with V30_STI.Shoot dry weight (*r* = 0.29***) at the early vegetative stage, V45_STI.Shoot dry weight (*r* = 0.23*) and V45_STI.Leaf injury (*r* = – 0.24*) at the late vegetative stage (Fig. 2). We independently ranked the top thirty tolerant genotypes based on V30_STI.Shoot dry weight at the early vegetative stage, V45_STI. Shoot dry weight at the late vegetative stage and STI. Seed yield at the reproductive stage (Supplementary Table S9). The growth stage comparison revealed nine tolerant genotypes in common between the early and late vegetative stages, thirteen between the early vegetative and reproductive stages, and eight between the late vegetative and reproductive stages. Four genotypes—VI001244AG, VI002611AG, VI002672AG and VI000981BG—were tolerant at all three stages. Interestingly, four tolerant genotypes at the early vegetative and three tolerant genotypes at the late vegetative stage were among the most sensitive genotypes at the reproductive stage.

### Genetic diversity, population structure and linkage disequilibrium

**Fig. 3:**
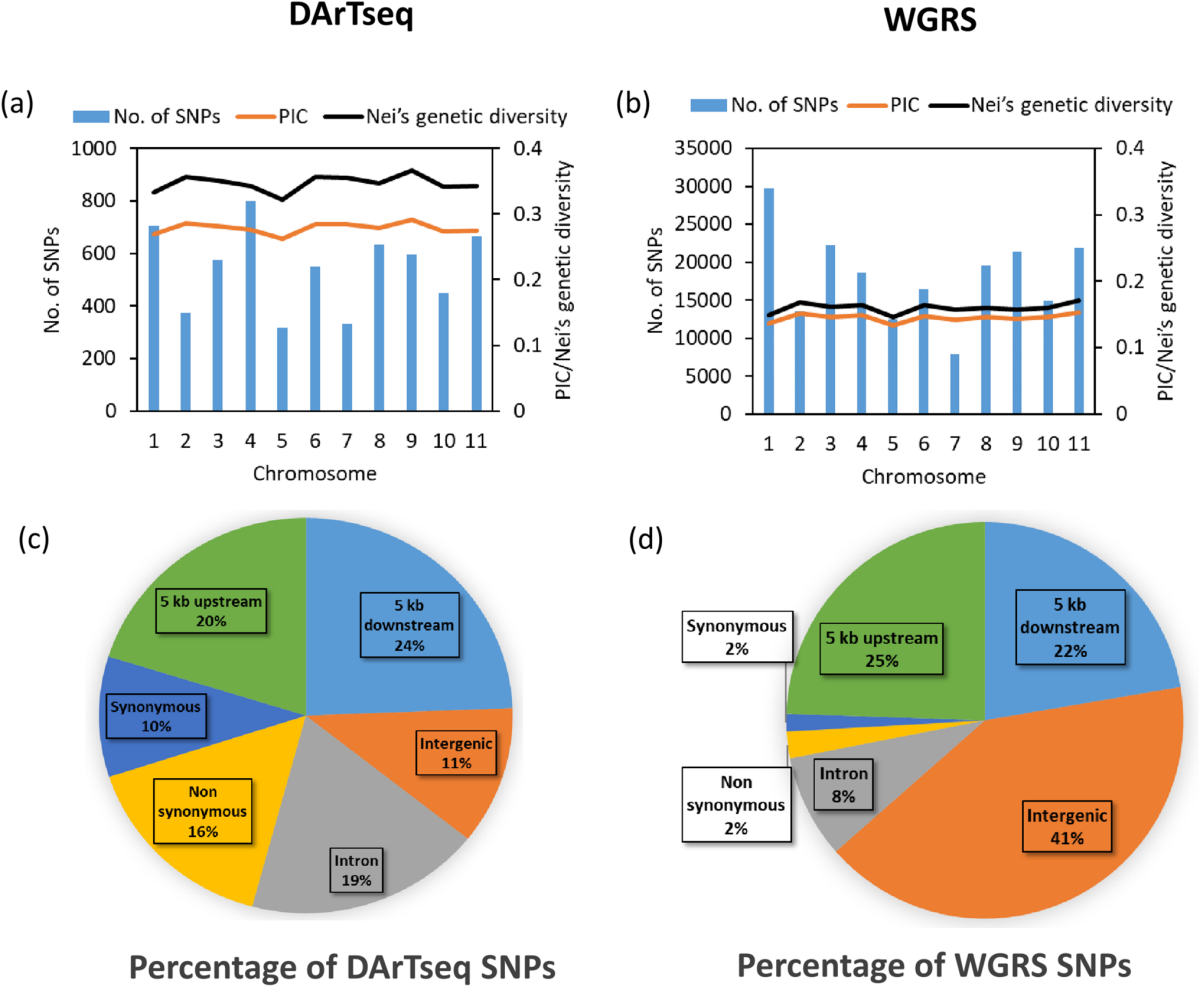
Distribution, polymorphism information content (PIC) and Nei’s genetic diversity of SNPs of different chromosomes of DArTseq SNPs (**a**) and WGRS SNPs (**b**). Annotation and percentage of SNPs at the whole-genome level of 5,991 DArTseq SNPs (**c**) and 198,474 WGRS SNPs (**d**)

A total of 5,991 DArTseq and 198,474 WGRS SNPs were distributed unevenly among the eleven mungbean chromosomes. The average number of SNPs per chromosome was 545 and 18,043, and the average distance between consecutive SNPs was 86.46 Kb and 2.64 Kb for DArTseq and WGRS sets, respectively (Supplementary Table S10 and Fig. 3a, b). Among the DArTseq SNPs, only 11% of SNPs were located in intergenic regions, 20% 5 kb upstream and 24% 5 kb downstream. The remaining 45% (2,644) of SNPs were located in genic regions. Among the 2644 genic SNPs, 1107 SNPs (19% of total DArTseq SNPs) were in introns, 590 (10% of total DArTseq SNPs) were synonymous SNPs, and 947 (16% of total DArTseq SNPs) were non-synonymous SNPs (Fig. 3c). In contrast, among the 198,474 WGRS SNPs, those located in intergenic regions accounted for the highest proportion (41%), followed by those in 5 kb upstream (25%) and 5 kb downstream (22%) regions. Only 12% (24,096) of SNPs were in genic regions. Among the 24,096 genic SNPs, most (16,861) were in introns, 2872 (1% of total WGRS SNPs) were synonymous SNPs, and 4363 (2% of total WGRS SNPs) were non-synonymous SNPs (Fig. 3d). PIC values were, on average 0.28, for DArTseq SNPs and 0.14 for WGRS SNPs. Similarly, Nei’s genetic diversity were, on average, 0.35 for DArTseq SNPs and 0.16 for WGRS SNPs (Fig. 3a, b).

**Fig. 4:**
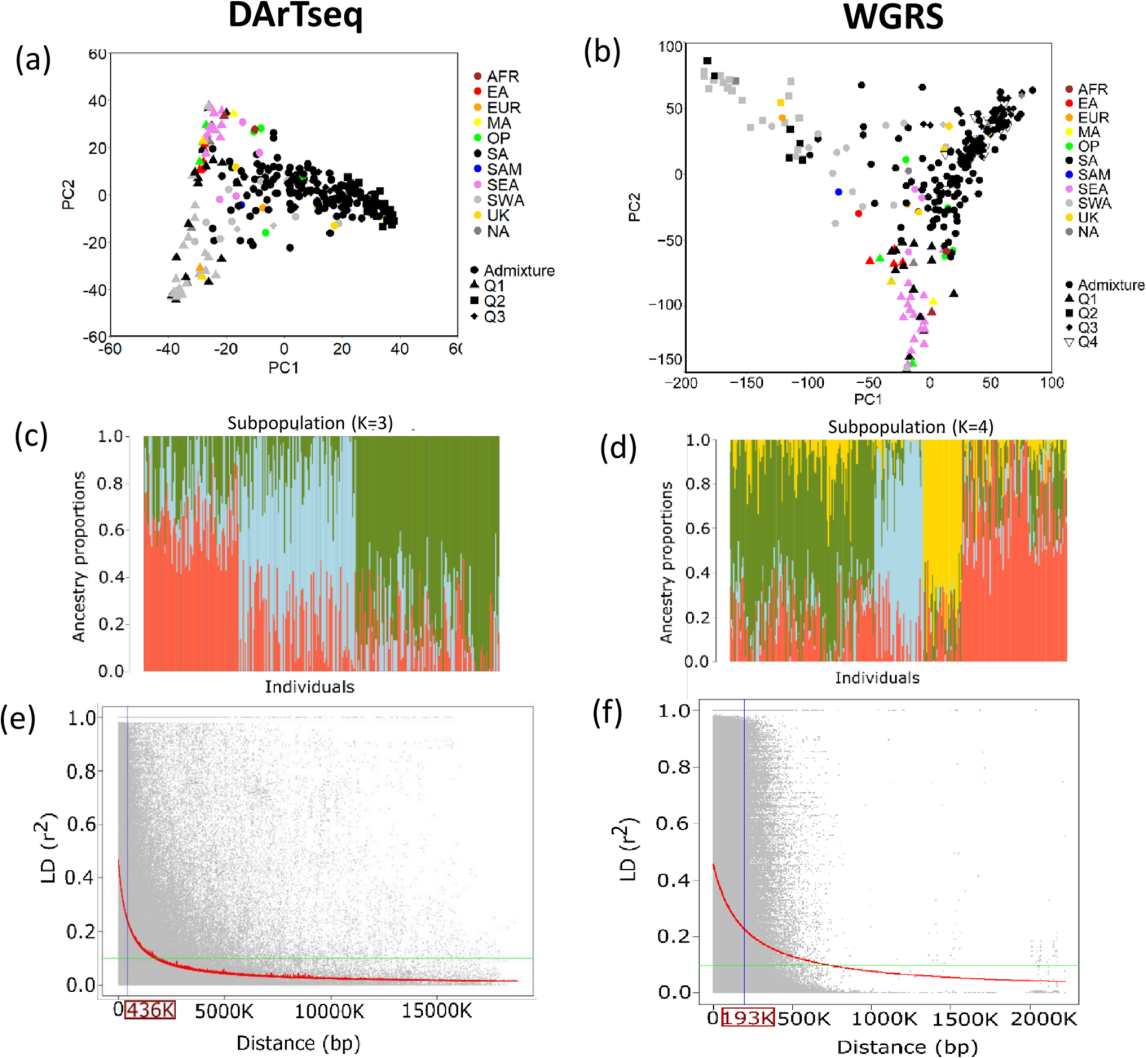
Principal component analysis of DARTseq SNPs (**a**) and WGRS SNPs (**b**) showing the subpopulation (cluster coefficients ≥ 70%) with geographical origin. Different shapes represent population structure groups, and colours represent geographical origin. Origin: AFR: Africa; EA: East Asia; EUR: Europe; MA: Central America; NA: North America; OP: Oceania and the Pacific; SA: South Asia; SAM: South America; SEA: Southeast Asia; SWA: South West Asia; UK: unknown. Population structure based on Q-matrix coefficients of DArTseq SNPs (**c**) and WGRS SNPs (**d**). Each colour segment represents a subpopulation. Linkage disequilibrium (r^2^) across the mungbean genome plotted against the physical distance of the SNPs. Left plot derived from DArTseq SNPs (**e**), and right plot derived from WGRS SNPs (**f**) of 292 mungbean mini-core collection genotypes

PCA was used to estimate and visualise the population structure among the mungbean mini-core genotypes independently using DArTseq and WGRS SNPs (Fig. 4a, b). The scree plot showed that the variance explained by the eigenvalue of each PC rapidly dropped after the first three PCs for DArTseq SNPs and after the first four PCs for WGRS SNPs (Supplementary Fig. S2a, b). The elbows in the scree plots therefore indicated that there were approximately three distinct subpopulations (*K* = 3) based on DArTseq SNPs and four distinct subpopulations (*K* = 4) based on WGRS SNPs within the mungbean mini-core genotypes, which was in agreement with the structure analysis (Fig. 4c, d). When considering the geographical origins of the genotypes, the results indicated that the subpopulation group corresponded to specific regions of origin (Fig. 4a, b and Supplementary Fig. S3: Neighbour-joining tree). For the DArTseq SNPs, South Asia (SA) genotypes were found across all subpopulations but predominantly in subpopulations 2 and 3. Genotypes from Africa, East Asia, Europe, North America, Oceanic Pacific, Southeast Asia and Southwest Asia were clustered in subpopulation 1 (Fig. 4a). For the WGRS SNPs, South Asian genotypes were predominantly in subpopulations 3 and 4. The WGRS SNPs data set further subdivided South West Asian genotypes and South East Asian genotypes, along with some other regions, into subgroups 1 and 2 (Fig. 4b). Over the whole genome, LD decayed at an average distance of 436 Kb within the DArTseq dataset and 193 Kb within the WGRS SNP dataset (Fig. 4e, f). The SNP coverage in both genotype datasets was dense enough relative to the more stringent LD decay distance estimated (193 Kb) and was therefore considered acceptable for GWAS analysis of in our study.

### Association mapping

GWAS was performed independently using the DArTseq and WGRS SNP markers together with the salinity tolerance indices of various phenotypic traits for 124 mungbean mini-core genotypes at the early vegetative and reproductive stages, and 292 genotypes at the late vegetative stage.

#### Early vegetative stage

**Fig. 5:**
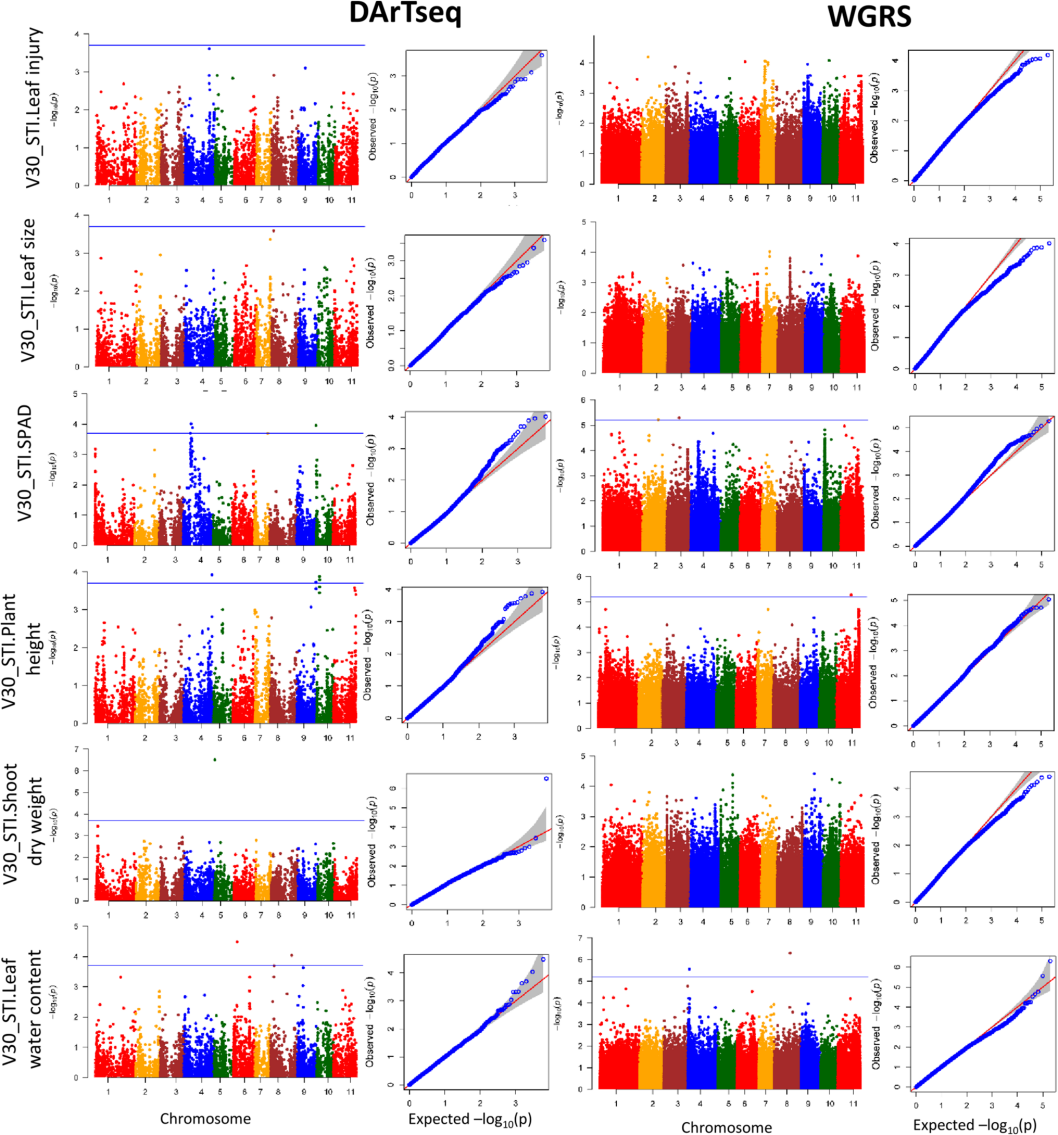
Manhattan plot and QQ plot of various traits at the early vegetative stage (30 DAS). The *x*-axis indicates the SNP location along the 11 mungbean chromosomes and the *y*-axis represents − log10(p) for the *p*-value of the marker–trait association. The blue horizontal line indicates the significance threshold (*P* < 1.89 × 10^−4^ for DArTseq SNPs and *P* < 5.03 × 10^−6^ for WGRS SNPs) (colour figure online)

Thirteen DArTseq SNPs and five WGRS SNPs were significantly associated with V30_STI.SPAD, V30_STI.Plant height, V30_STI.Shoot dry weight and V30_STI.Leaf water content. Importantly, none of the associations were common to multiple traits: each trait appeared to have its own unique set of SNP marker associations. The eighteen significantly associated SNPs were distributed across all chromosomes except Chr.1 and individually accounted for 5.8–16.3% of the phenotypic variance (Fig. 5 and Supplementary Table S11). The highest number of significant SNPs (five) on a single chromosome was on Chr.4, followed by three SNPs on Chr.8 and Chr.10, and one SNP each on Chr.2, Chr.3, Chr.5, Chr.7, Chr.8, Chr.9 and Chr.11. Interestingly, the DArTseq dataset had significant associations in different genomic regions compared to the WGRS dataset: no common genomic region of interested was identified by both genotype datasets. Among the entire eighteen significant SNPs, only one SNP on Chr.5 for V30_STI.Shoot dry weight exceeded the FDR *P*-value < 0.05, with the remaining seventeen SNPs significant at the less stringent *P*-value thresholds of 1.89 × 10^−4^ for the DArTseq SNPs and 5.03 × 10^−6^ for the WGRS SNPs.

**Fig. 6:**
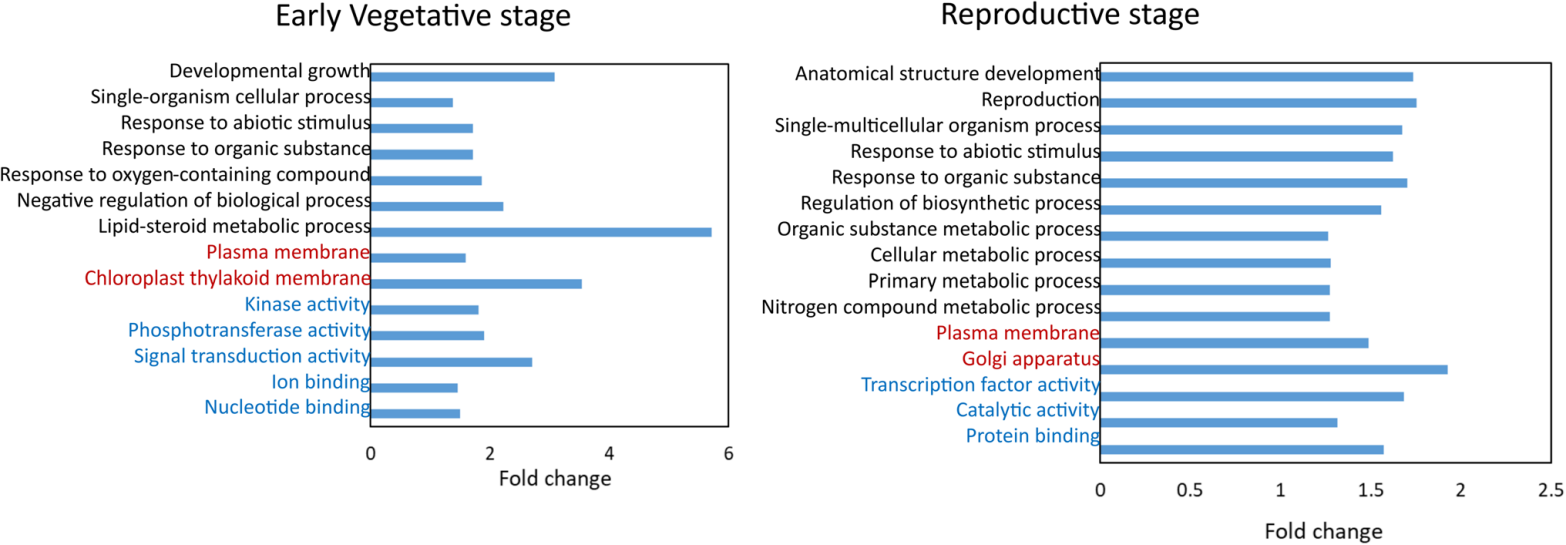
Ontology enrichment analysis of genes (Arabidopsis orthologues) near significant SNPs. Over-represented functional categories occurred at the early vegetative and reproductive stages. Enrichment presented in fold-change (percentage of enriched GO terms in salinity stress compared to percentage of reference GO terms in mungbean genome). The coloured text indicates the ontology source: black for biological processes, red for cellular components, and blue for molecular function (colour figure online)

A total of 404 positional candidate genes (336 for DArTseq SNPs and 38 for WGRS SNPs) were found within the 193 Kb LD decay windows flanking these significant SNPs (Supplementary Tables S12 and 13). The GO enrichment analysis of Arabidopsis orthologues for the 404 candidate genes revealed different functional categories response to abiotic stimulus, oxygen containing compound, developmental growth were over-represented for salinity stress (Fig. 6). To mine functional candidate genes, we investigated three candidate genes that contained significant SNPs within the coding region and caused non-synonymous mutations (Table 2). Chr10.51, encoding an ethylene receptor, contained a SNP that explained 10.7% of the phenotypic variance for V30_STI.SPAD (Table 2). The SNP corresponded to a missense mutation with a C/G substitution and corresponding H/D amino acid change, with a significant difference in V30_STI.SPAD observed between allele genotypes (Supplementary Fig. S4a). Chr7.1696, encoding callose synthase, contained a nonsense SNP on Chr.7 associated with V30_STI.SPAD and explained 12.4% of the phenotypic variance (Table 2). The corresponding SNP substitution (C/T) stopped the function of a Q amino acid, with a significant difference in V30_STI.SPAD between allele genotypes (Supplementary Fig. S4b). Lastly, the Dynamin-related protein 5A isoform protein encoded by Chr6.465 was associated with V30_STI.Leaf water content, explaining 11.6% of phenotypic variance (Table 2). The SNP on Chr.6 produced a missense mutation involving a C/G nucleotide substitution (C/G) and an L/V amino acid change, with a significant difference in V30_STI.Leaf water content between allele genotypes (Supplementary Fig. S4c).

**Table 2:** Candidate genes containing significant SNPs that cause missense or nonsense mutation. *P*-value, minor allele frequency (MAF), phenotypic variance explained (PVE), allele and functional annotation of the candidate genes

Trait	SNP ID (Chromosome name with position)	*P*-value	MAF	PVE (%)	Allele	Candidate Gene ID	Functional annotation
V30_STI.SPAD	DArT_7.25495308	2.0 × 10^–4^	0.32	12.4	C/T	Chr7.1696	Callose synthase
	DArT_10.1082331	1.1 × 10^–4^	0.22	10.7	C/G	Chr10.51	Ethylene receptor
V30_STI.Leaf water content	DArT_6.7112220	3.3 × 10^–5^	0.42	11.6	C/G	Chr6.465	Dynamin-related protein
V45_STI.Plant height	WGRS_8.40300398	6.0 × 10^–6^	0.05	18.4	G/A	Chr8.2689	GTP diphosphokinase
V45_STI.Shoot dry weight	DArT_11.41691382	5.1 × 10^–8^*	0.45	10.6	C/T	Chr11.2213	Cytochrome P450
STI.Time to maturity	DArT_3.27953227	2.0 × 10^–9^*	0.28	9.0	A/G	Chr3.1618	bHLH -type transcription factor
STI.No of pods/plant	DArT_11.35419254	7.3 × 10^–5^	0.16	7.3	G/A	Chr11.1653	Kinesin-10-type motor protein
STI.Seed yield	DArT_11.35419254	7.3 × 10^–5^	0.16	10.7	G/A	Chr11.1653	Kinesin-10-type motor protein

^*^Indicates significance at false discovery rate (FDR) threshold at 5% significance level

#### Late vegetative growth stage

**Fig. 7:**
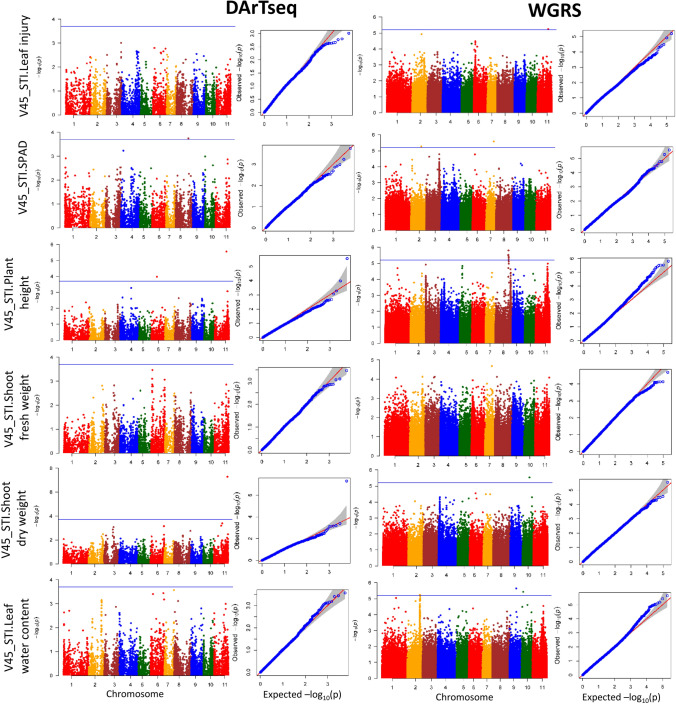
Manhattan plot and QQ plot of various traits at the late vegetative stage (45 DAS). The *x*-axis indicates the SNP location along the 11 mungbean chromosomes, and the *y*-axis represents − log10(p) for the *p*-value of the marker–trait association. The blue horizontal line indicates the significance threshold (*P* < 1.89 × 10^−4^ for DArTseq SNPs and *P* < 5.03 × 10^−6^ for WGRS SNPs) (colour figure online)

At the late vegetative stage, four DArTseq SNPs and fourteen WGRS SNPs were significantly associated with V45_STI.Leaf injury, V45_STI.SPAD, V45_STI.Plant height, V45_STI.Shoot dry weight and V45_STI.Leaf water content. These SNPs were distributed on Chr.2, Chr.6, Chr.7, Chr.8, Chr.9, Chr.10 and Chr.11 and individually accounted for 0.3–25.0% of the phenotypic variation in traits (Fig. 7 and Supplementary Table S11). As with the early vegetative stage, no common genomic regions were identified either between the different traits or between the DArTseq and WGRS GWAS analyses. The highest number of SNPs (eight) on a single chromosome occurred on Chr.8, followed by three SNPs on Chr.11, two SNPs on Chr.2 and Chr.10, and one SNP each on Chr.6, Chr.7 and Chr.9. Among the eighteen significant SNPs, only two SNPs on Chr.11 for V45_STI.Plant height and V45_STI.Shoot dry weight exceeded the FDR *P*-value < 0.05, while the remaining sixteen SNPs were significant at the less stringent *P*-value thresholds of 1.89 × 10^−4^ for the DArTseq SNPs and 5.03 × 10^−6^ for the WGRS SNPs.

A total of 237 positional candidate genes (79 for DArTseq SNPs and 158 for WGRS SNPs) occurred within the 193 Kb LD decay windows flanking the significant SNPs (Supplementary Tables S12 and 13). The GO enrichment analysis of Arabidopsis orthologues for these 237 genes revealed that no significant functional categories were over-represented under salinity stress. To mine functional candidate genes, we investigated two candidate genes that contained significant SNPs within the coding region and caused non-synonymous mutations (Table 2). The first of these genes was Chr8.2689, which encodes guanosine tetra phosphate (GTP) diphosphokinase on Chr.8 and was associated with V45_STI.Plant height. The missense SNP in this gene, which comprised an A/G substitution (A/G) with an S/L amino acid change, explained 18.4% of the phenotypic variance (Table 2) and led to a significant difference in V45_STI.Plant height between allele genotypes (Supplementary Fig. S4d). Meanwhile, a significant SNP on Chr.11 associated with V45_STI.Shoot dry weight was mapped to an LD block of 9 Kb length (11:41,681,709–41691411) containing three SNPs (Supplementary Fig. S5a, b). This block explained 19.6% of phenotypic variance and harboured three genes, Chr11.2212, Chr11.2213 and Chr11.2214, all encoding Cytochrome P450 family genes (Supplementary Table S12). Among the three SNPs, only one was located in an exon of Chr11.2213 and caused a missense mutation. An SNP substitution (C/T) caused a G/S amino acid change, corresponding to a significant difference in V45_STI.Shoot dry weight between allele genotypes (Supplementary Fig. S5c).

#### Reproductive stage

**Fig. 8:**
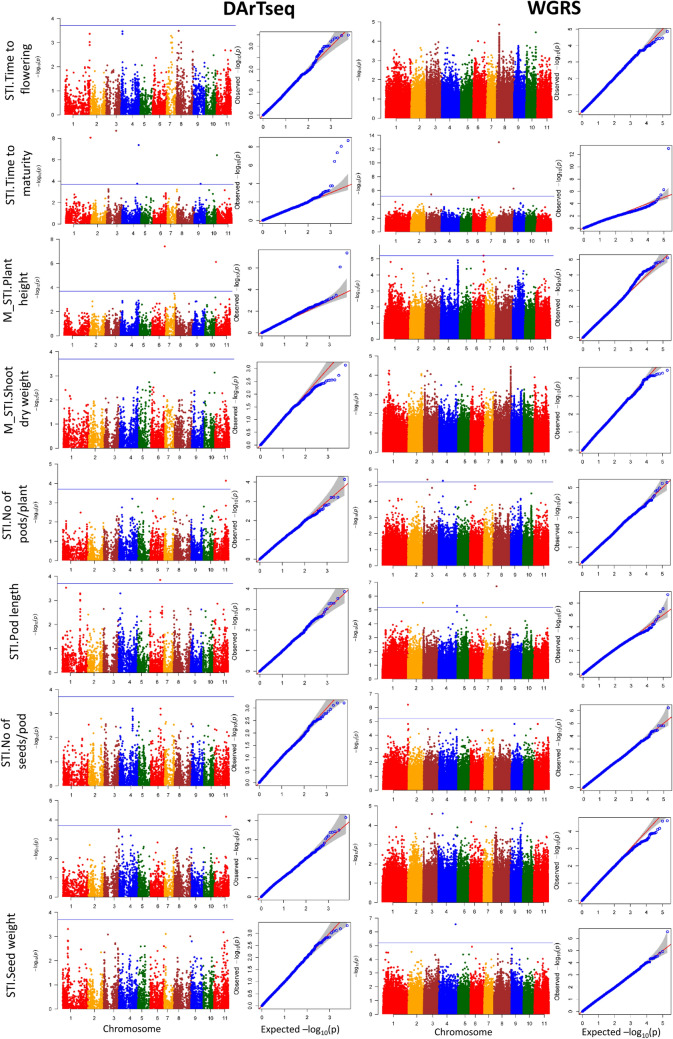
Manhattan plot and QQ plot of various traits at the reproductive stage. The *x*-axis indicates the SNP location along the 11 mungbean chromosomes, and the y-axis represents − log10(p) for the *p*-value of the marker–trait association. The blue horizontal line indicates the significance threshold (*P* < 1.89 × 10^−4^ for DArTseq SNPs and *P* < 5.03 × 10^−6^ for WGRS SNPs) (colour figure online)

Turning to the reproductive stage of growth, eleven DArTseq SNPs and eleven WGRS SNPs were significantly associated with seven phenotypic traits reflecting salinity tolerance during reproduction, including STI.Time to maturity, M_STI.Plant height, STI.No of pods/plant, STI.Pod length, STI.No of seeds/pod, STI. Seed yield and STI.Seed weight. These significant SNP associations were distributed on all chromosomes except Chr.5 and Chr.7, and accounted for 3.4–25.8% of phenotypic variance per trait (Fig. 8 and Supplementary Table S11). No common significant genomic regions were identified across the DArTseq and WGRS GWAS analyses. One SNP on Chr.11 significantly correlated with STI. No of pods/plant and STI.Seed yield. The highest number of SNPs per chromosome (five) occurred on Chr.4, followed by three SNPs on Chr.3, Chr.6, Chr.8 and Chr.11, two SNPs on Chr.1 and one SNP each on Chr.2, Chr.9 and Chr.10. Among the twenty two significant SNPs, five SNPs on Chr.1, Chr.3, Chr.4, Chr.8 and Chr.10 associated with STI.Time to maturity, two SNPs on Chr.6 and Chr.11 associated with M_STI.Plant height, and one SNP on Chr.8 associated with STI.Pod length exceeded the FDR P-value threshold, while the remaining fourteen SNPs were significant at the less stringent P-value threshold of 1.89 × 10^−4^ for DArTseq SNPs and 5.03 × 10^−6^ for WGRS SNPs.

**Fig. 9:**
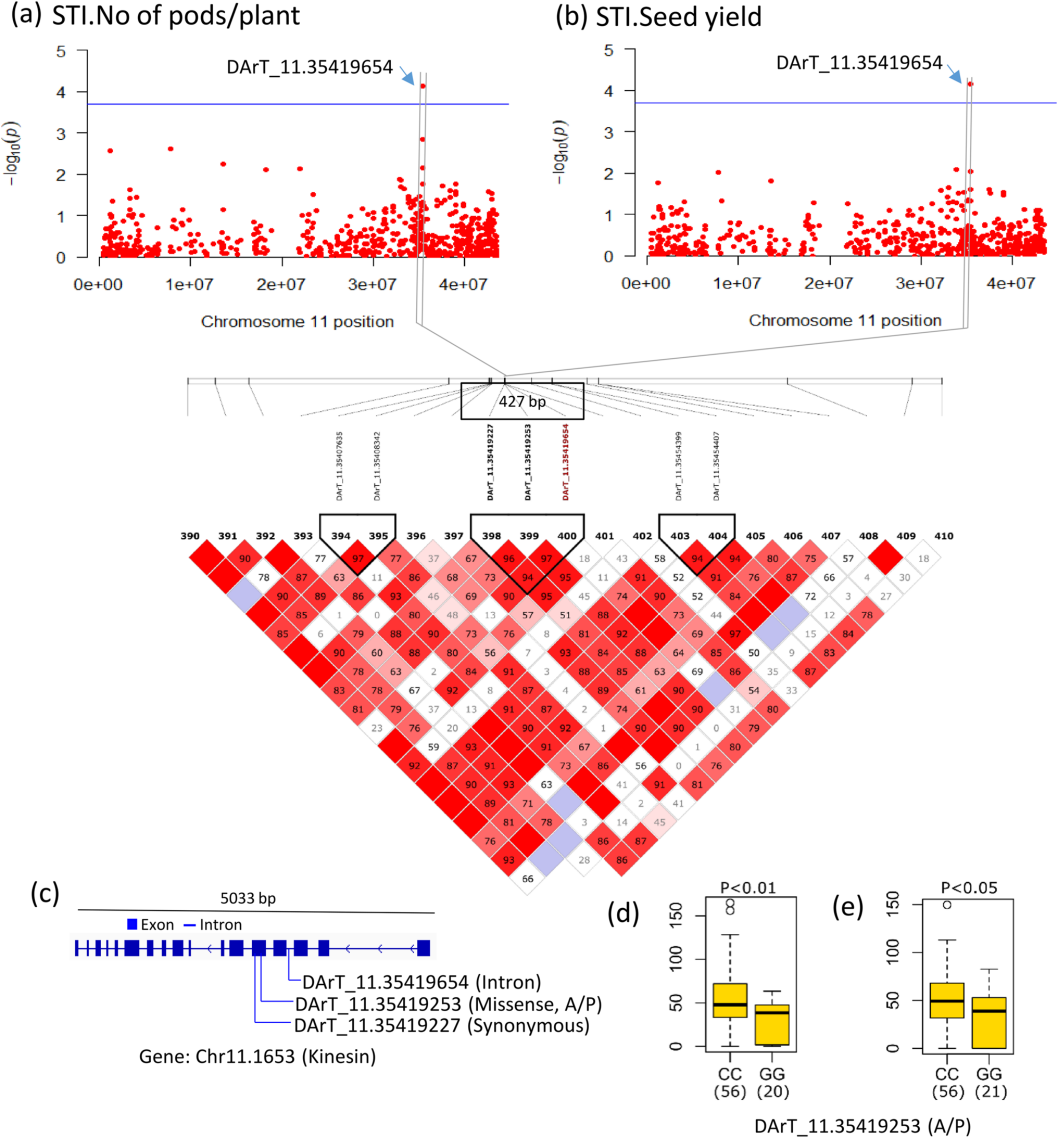
Local Manhattan plot on chromosome 11 for STI.No of pods/plant (**a**) and STI.Seed yield (**b**) showing significant markers. LD heatmap surrounding the significant SNP on chromosome 11 showing three SNPs in the candidate region. Each coloured diamond shows correlations between the two markers. Horizontal blue line indicates the significance threshold (*P*-value < 1.89 × 10^−4^). Exon–intron structure of candidate gene Chr11.1653 and location of SNPs (**c**). Boxplots for the missense SNP DArT_11.35419253. Genotypes were divided into two groups at each locus based on allele type. Significant differences between STI.No of pods/plant (**d**) and STI.Seed yield (**e**) of these two allele groups were analysed by *t*-test (*P* < 0.05). The number of genotypes harbouring the corresponding allele is shown in brackets

A total of 707 positional candidate genes (318 for DArTseq SNPs and 389 for WGRS SNPs) occurred in the 193 Kb LD decay windows flanking the significant SNPs (Supplementary Tables S12 and 13). The GO enrichment analysis of the corresponding Arabidopsis orthologues showed that different functional categories including anatomical structure development, reproduction, response to abiotic stimulus were over-represented for salinity stress (Fig. 6). One candidate gene, Chr3.1618 (encoding a bHLH-type transcription factor) associated with STI.Time to maturity contained a missense mutation (Table 2). This SNP substitution (A/G) corresponded to an M/T amino acid change, but no significant difference in STI.Time to maturity occurred between the two allele genotypes (Supplementary Fig. S4e). One SNP on Chr.11 associated with two highly correlated traits, STI.No of pods/plant and STI.Seed yield (Fig. 9a, b), was mapped to a 427 bp length LD block (11:35,419,227–35,419,654) containing three SNPs (Fig. 9c), one of which was located within an intron region, and two were located within exonic regions of a gene (Chr11.1653) encoding Kinesin-10-type motor protein (Fig. 9d). One of these markers within the coding sequence caused a missense mutation involving a C/G nucleotide substitution and a corresponding A/P amino acid change significantly associated with differences for STI.No of pods/plant and STI.Seed yield (Fig. 9e, f).

Comparing the GWAS results, we did not find any significant SNPs common to multiple traits across the early vegetative, late vegetative and reproductive stages (Supplementary Table S11). However, we found eight significant SNPs located on Chr.11, spanning from 28,048,656 to 41,691,382 bp (13 Mb), associated with multiple traits: V30_STI.Plant height at the early vegetative stage, V45_STI.Leaf injury, V45_STI.Plant height and V45_STI.Shoot dry weight at the late vegetative stage, and STI.No of pods/plant and STI.seed yield at the reproductive stage.

### Genomic prediction

**Fig. 10:**
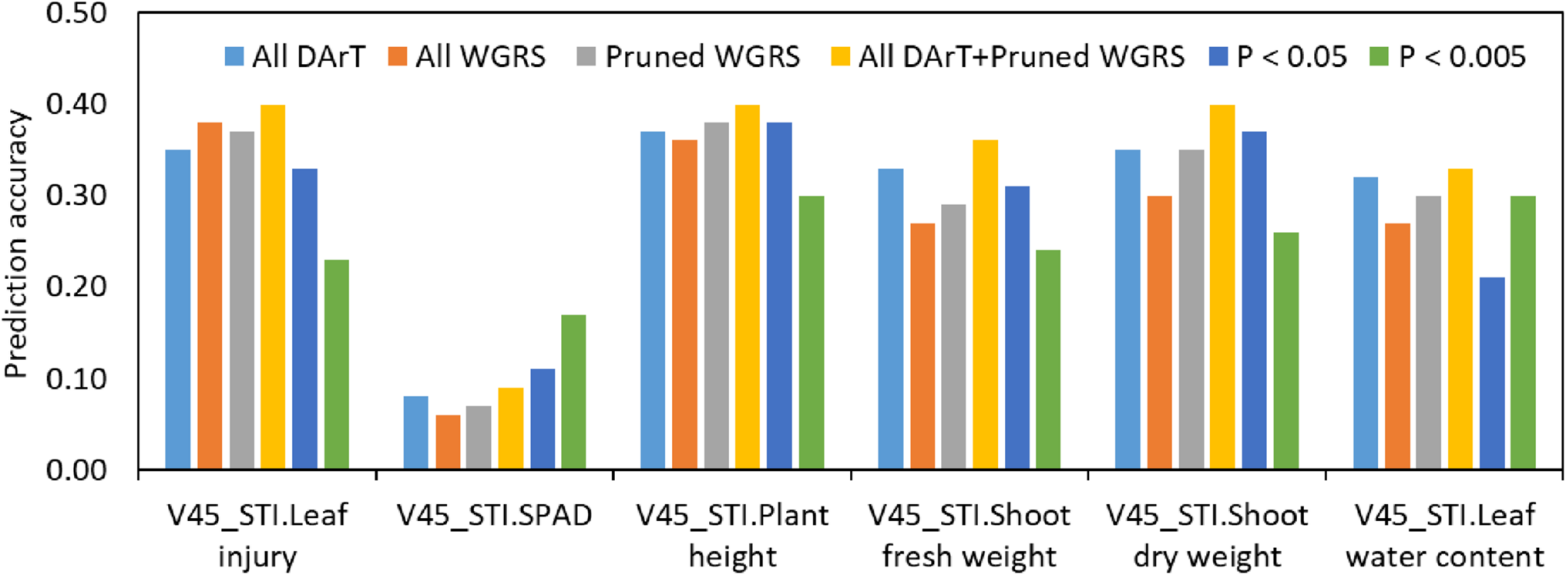
Genomic prediction accuracies for different traits at the late vegetative stage

The GP model fitted with all DArTseq SNPs showed comparatively higher prediction accuracy (on average 0.30) than the all WGRS SNPs (on average 0.27) for most of the traits except V45_STI.Leaf injury (Fig. 10). Using the pruned WGRS SNPs slightly improved prediction accuracies (on average 0.29) for most of the traits than the using all WGRS SNPs but none of the prediction accuracies performed better than the DArTseq SNPs. However, combining the DArTseq and pruned WGRS SNPs improved prediction accuracies for all of the traits and showed 0.33–0.40 prediction accuracies for most of the traits except 0.09 in V45_STI.SPAD. Inclusion of GWAS results, GP model using SNPs with *P* value < 0.05 showed the nearest prediction accuracy (on average 0.29) to the others models for most of the traits. However, GP model using SNPs with *P* value < 0.005 increased prediction accuracy to 0.17 for V45_STI.SPAD and showed comparable accuracy for V45_STI.Leaf water content while reducing prediction accuracy for other traits.

## Discussion

Mungbean is considered a salt-sensitive species and crops exposed to salinity at one or more growth stages incur a severe yield penalty. To mitigate the adverse effect of salinity stress, it is necessary to breed cultivars with salinity tolerance at different growth stages, including the reproductive stage, which is particularly prone to stress. This study’s systematic approach using a mungbean mini-core germplasm collection revealed an extensive range of genetic variation for salinity tolerance in mungbean at the early vegetative, late vegetative and reproductive stages. The extent of this genetic variation contrasts with previous studies (Sehrawat et al. 2014a; Manasa et al. 2017; Iqbal et al. 2024b), which comprised smaller numbers of local germplasm and breeding lines. An apparent inconsistency was found for salinity tolerance between the three growth stages, indicating that distinct mechanisms control salinity tolerance in mungbean at the vegetative and reproductive stages. Linking the phenotypic data with two sources of genotypic markers derived from DArTseq and WGRS approaches revealed important genomic regions and candidate genes associated with salinity tolerance at the three growth stages. Comparison between the low-density DArTseq SNP markers and high-density WGRS SNPs showed that a higher density of markers did not improve association mapping precision or GP accuracy for salinity tolerance. This study is the first to investigate the genetic basis of salinity tolerance in mungbean across vegetative and reproductive stages, and also the first to evaluate the potential of GP for this trait.

### Phenotyping, GWAS and genomic prediction of salinity tolerance in mungbean

Salinity tolerance is a complex phenomenon regulated by multiple factors. This study highlights the importance of phenotyping for salinity tolerance at various developmental stages to develop salt-tolerant mungbean varieties with enhanced plant resilience. We found a weak correlation between the salinity tolerance index of various traits at the early vegetative, late vegetative and reproductive stages, consistent with reports on chickpea (Vadez et al. 2007), rice (*Oryza sativa* L.; Chen et al. 2022a) and barley (Saade et al. 2020). We also compared our results with previously reported germination percentages for the same mini-core collection grown in 50 mM NaCl (Breria et al. 2020b). We found no significant relationships between salinity tolerances at the germination stage and later growth stages, with salt-tolerant genotypes at early and/or late vegetative stages not always salt-tolerant at the reproductive stage. These results demonstrate the genetic complexity of salinity tolerance in mungbean and highlight the importance of evaluating salinity tolerance at multiple growth stages to screen mungbean germplasm for seed production under salinity stress. Moreover, we found only four genotypes (VI001244AG, VI002611AG, VI002672AG and VI000981BG) that exhibited salt tolerance at all three growth stages. The consistent performance of these four genotypes across all growth stages highlights their potential as key genetic resources or donor parents for breeding programs of improving salinity tolerance in mungbean. Since the salinity screening experiment was conducted in a temperature-controlled glasshouse environment, the results must be validated by growing a range of salt-tolerant and sensitive genotypes in multi-location field trials under saline conditions.

The GWAS analyses of the salinity tolerance index for twenty one traits using DArTseq- and WGRS-derived SNPs revealed fifty eight significantly associated SNPs, but none were common to the early vegetative, late vegetative and reproductive stages. This outcome is consistent with the weak phenotypic correlations observed across growth stages and further supports the existence of different salinity tolerance mechanisms at different growth stages. This study is the first to report association or QTL mapping for salinity tolerance in mungbean at the vegetative and reproductive stages. Other studies have reported association or QTL mapping for salinity tolerance during germination and early seedling growth (Breria et al. 2020b; Liu et al. 2022) and other abiotic stresses (waterlogging, Kyu et al. 2024). Breria et al. (2020b) identified two genomic regions associated with germination percentage, and Liu et al. (2022) found seven significant associations with seedling survival rate after salt treatment for 10 and 15 days. Of the fifty eight significant marker associations in this study, at the early vegetative stage eighteen SNPs were associated with four traits in eighteen genomic regions, at the late vegetative stage eighteen SNPs were associated with five traits in fifteen genomic regions, and at the reproductive stage twenty-two SNPs were associated with seven traits in twenty genomic regions. The availability of multiple marker associations at each developmental stage provides confidence for developing markers without bias towards a particular growth stage, which will help develop tolerant varieties throughout their life cycle. Furthermore, we identified eight significant SNPs located on Chr. 11, spanning in a 13 Mb genomic regions, associated with multiple traits across the three growth stages, indicating that Chr.11 may harbour key loci for broad-spectrum salinity tolerance in mungbean. These loci would be promising targets for further validation and functional characterisation, and their conversion into cost-effective KASP markers could facilitate marker-assisted breeding for salinity across multiple growth stages.

In addition to the potential benefits of the GWAS analysis from a practical perspective, this study has also revealed insights into the genetic architecture of salinity stress regulation in mungbean. A total of 404 positional candidate genes at the early vegetative stage, 237 positional candidate genes at late vegetative stage and 707 positional candidate genes at the reproductive stage were found in the 193 Kb LD decay windows flanking the significant SNPs. Among them, seven candidate genes containing non-synonymous SNPs within the coding sequence were of particular interest for further investigation within the literature, as these candidate genes may play an important role in salinity tolerance in mungbean by changing protein structure. At the early vegetative stage, Chr7.1696—associated with V30_STI.SPAD—encodes 1,3-beta-glucan synthase involved in callose synthase at the plasma membrane of plant cells (Drábková and Honys 2017). Solute and water transport can occur in plants through apoplastic or cell-to-cell (transmembrane and symplastic) pathways. Symplastic transport occurs through the plasmodesmata, and callose deposition near the neck zone of plasmodesmata controls solute permeability (Wu et al. 2018). Regulation of callose deposition in response to salinity stress has been reported in Arabidopsis (Hunter et al. 2019) and barley (Ho et al. 2020). Chr10.51—associated with V30_STI.SPAD—encodes an ethylene receptor related to ethylene signalling (Gallie 2015). Ethylene signalling plays a vital role in plant salinity tolerance by maintaining Na^+^/K^+^ homeostasis and ROS by inducing antioxidant enzymes (Tao et al. 2015; Riyazuddin et al. 2020). Chr6.465—associated with V30_STI.Leaf water content—encodes dynamin-related protein 5A isoform X2. The Arabidopsis dynamin-related protein family comprises 16 proteins and controls intracellular vesicular trafficking that regulates the movement of small molecules and nutrients across the plasma membrane by solute transporters (Baral et al. 2015). Its function(s) in relation to salinity tolerance has not been well studied.

At the late vegetative stage, Chr8.2689—associated with V45_STI.Plant height—encodes GTP diphosphokinase involved in the guanosine tetraphosphate biosynthetic process. Guanosine tetraphosphate is associated with stress signalling, acting as a conserved regulator of photosynthetic activity in chloroplasts (Mehrez et al. 2023). Under salinity stress, guanosine tetraphosphate synthesis likely participates in the transcriptional induction of several stress-responsive genes in Arabidopsis (Yamada et al. 2003); however, its function in crop salinity tolerance has not been well reported. Chr11.2213—associated with V45_STI.Shoot dry weight—encodes cytochrome P450 family proteins. The cytochrome P450 gene family is reportedly involved in salinity tolerance through ROS homeostasis and hormone signalling (Magwanga et al. 2019; Wang et al. 2019; Pandian et al. 2020) and regulating apoplastic barrier formation in roots (Krishnamurthy et al. 2020).

At the reproductive stage, Chr3.1618—associated with STI.Time to maturity—encodes a bHLH-type transcription factor. The basic helix-loop-helix (bHLH) transcription factors (TFs) play a critical role in various physiological processes, such as plant development, secondary metabolism and abiotic stress responses (Guo et al. 2021). The overexpression of a bHLH TF (AtMYC2) gene conferred increased salinity tolerance in Arabidopsis by increasing proline concentration and osmotic adjustments (Verma et al. 2020). Chr11.1653—associated with STI.No of pods/plant and STI.Seed yield—encodes the Kinesin-10-type motor protein. Kinesins are motor proteins that can move through microtubules and regulate the long-distance transport of various cellular components (Nebenführ and Dixit 2018). The Kinesin-10 subfamily may contribute to the delivery of Golgi vesicles in phragmoplasts (Lee et al. 2001). Some Kinesin subfamily members reportedly regulate microtubule organisation and ionic homeostasis under salinity stress (Chen et al. 2022b; Gu et al. 2023). However, the function of the Kinesin-10 subfamily in salinity tolerance remains unknown. We were also interested in the candidate genes involved in ion channels and transporters in relation to salinity tolerance. In brief, we found two cation channels, six cation transporters, one H^+^/Na^+^ cation antiporter (NHX), one anion channel and four anion transporters (Table S14). Thus, our GWAS identified candidate genes with plausible potential functional roles, strengthening our understanding of the genetic pathways regulating salinity tolerance in mungbean. However, it should be cautioned that the identified markers and candidate genes require experimental validation to confirm their functional roles and applicability in breeding. Future research should focus on validating these markers and candidate genes in genetically diverse populations under field conditions and by integrating multi-omics approaches and functional studies, such as transcriptomic validation or CRISPR-based gene editing.

Interestingly, we unexpectedly found similar numbers of SNP markers derived from DArTseq (28 markers) and WGRS (30 markers) genotyping methods significantly associated with salinity stress traits. We also did not find any common genomic regions for a particular trait using the two markers data sets. We hypothesised that high-density WGRS SNPs would detect more significant associations with higher precision than low-density DArTseq SNPs. The comparison of genomic predictive abilities between the two marker data sets for six traits at the late vegetative stage also revealed that WGRS SNPs did not increase the GP accuracy in most traits. In a similar study, 1.6 K DArTseq SNPs had better GP accuracies for yield and yield-related traits in chickpea than 89 K GBS SNPs (Roorkiwal et al. 2018). Similar to our results, high-density markers did not increase GP accuracy for most studied traits in wheat (Jiang et al. 2015; Ladejobi et al. 2019) and rapeseed (Werner et al. 2018).

There are plausible explanations for the observed lack of improvement in the power of GWAS and GP analyses using WGRS compared to DArTseq genotypic data. DArTseq is largely free from ascertainment bias as it simultaneously discovers and genotypes markers within the studied population using restriction enzyme-based complexity reduction, which preferentially targets gene-rich and repetitive regions (Jaccoud et al. 2001; Kilian et al. 2012; Cruz et al. 2013). Thus, despite producing markers at a lower density, DArTseq ensures unbiased and informative markers are discovered for trait association and GP in a cost-effective manner. Although WGRS provides a much higher density of SNPs, the presence of large numbers of less informative (low PIC value) SNPs in LD can reduce the detection of significant SNP associations in GWAS and affect GP accuracy (Wall et al. 2014; Lipka et al. 2015; van den Berg et al. 2017; Roorkiwal et al. 2018). Moreover, populations of limited size cannot capture the full advantage of high-density SNPs (Meuwissen 2009). For example, the high-density WGRS SNP dataset uncovered more significant marker–trait associations amongst 292 genotypes at the late vegetative stage than it did for the smaller panel of 124 genotypes during the early vegetative and reproductive stages in this study. Better precision might be achieved using high-density SNPs in larger populations, where important functional variants may be less rare. Lastly, we also used a subset of WGRS SNPs in our study and selected 5,917 SNPs based on marker quality (PIC > 0.26). The GP accuracy using these pruned WGRS SNPs revealed only slight differences in the prediction accuracy for most traits compared to using all markers. This result suggests that an extremely high-density markers may not necessarily be needed; rather, a smaller number of high-quality markers may be sufficient enough for analyses, provided that their density is no less than the estimated genome-wide LD decay distance. This advantageously offers a more cost-effective approach for genomic selection by reducing genotyping costs and computational demands.

To the best of our knowledge, we have reported the application of GP in mungbean for the first time. We found that combining all DArTseq and pruned WGRS SNPs improved prediction accuracy for all of the salinity tolerance traits. Combining the markers from different genotyping systems (the Infinium and GRAS-Di) similarly improved prediction accuracies for different fruit traits in apple (Minamikawa et al. 2024). These results suggest that as new genotyping data are continuously developed, the integration of data from different genotyping platforms would be useful to enhance genotypic coverage, improve accuracy and marker density. Using GWAS-based markers in GP models showed their potential for practical application in genomic selection. The inclusion of only GWAS-based markers (SNPs with *P* value < 0.05) in GP models showed comparable prediction accuracies to those obtained using the entire marker set for most traits, highlighting the potential of using GWAS-based markers in GP. Incorporating prior biological information from GWAS into GP has been shown to enhance or comparable prediction accuracy for complex traits in many crop species (Li et al. 2018; Messina et al. 2018; Peng et al. 2025). While the predictive abilities identified across the traits in our study were moderate (0.17–0.40), this can still facilitate genetic gain in breeding salinity tolerance in mungbean through reduced breeding cycle times. Bandillo et al. (2022) found that genomic prediction accuracies of 0.27–0.42 showed better performance than phenotypic selection for seed yield in soybean. Echoche et al. (2025) found up to 89% genetic gain compared to phenotypic selection alone, despite a modest predictive ability of 0.3 for growth-related traits in forage legumes, white clover (*Trifolium repens* L.). Overall, our results highlighted the potential of employing GP for salinity tolerance in mungbean. However, it should be noted that the prediction accuracy was assessed only through a cross-validation scheme. Further detail research is required to determine if these findings remain consistent when the approach is applied to selection candidates in practical breeding scenarios.

### Stage-specific responses and molecular adaptations to salinity stress in mungbean

Salinity stress affected mungbean growth in a stage-specific manner in this study. This observation is consistent with the two-phase salinity response model (Munns 1993), whereby (i) osmotic stress initially reduces water uptake, cell expansion, and leaf development, and (ii) ionic stress later leads to ion toxicity, impairing photosynthesis and reproductive function (Munns and Tester 2008). At the early vegetative stage (15 days after salinity treatment), growth reduction in mungbean was likely affected by osmotic stress. Early stress responses such as reduced biomass and leaf area are indicative of water limitations due to osmotic stress. The absence of severe leaf necrosis at this stage further supports this conclusion. Salinity stress at the vegetative stage increased leaf succulence (higher water content per unit leaf area), which is an adaptive response to dilute cellular salt concentrations (Munns et al. 2016). However, this also reduced leaf expansion, leading to smaller but thicker leaves with lower total photosynthetic capacity (Iqbal et al. 2024a). As salinity stress persisted at later growth stages, the cumulative effects of osmotic and ionic stress became evident, potentially due to excessive Na⁺ and Cl⁻ accumulation, as seen in other legumes like chickpea under salinity stress (Turner et al. 2013; Samineni et al. 2011; Khan et al. 2015). Toxic Na⁺ and Cl⁻ accumulation in chloroplasts impaired photochemical reactions, leading to declines in chlorophyll content (SPAD), net photosynthesis, and assimilate production (Khan et al. 2017; Kotula et al. 2019; Iqbal et al. 2024a). At the reproductive stage, seed yield declined by 45%, primarily due to reduced pod numbers (*r* = 0.9***) and 100-seed weight (*r* = 0.39***), suggesting that salinity limited assimilate availability for reproductive development (Khan et al. 2017; Iqbal et al. 2024b). Additionally, the reduction in seed yield significantly correlated with the reduction in shoot dry mass at the early and late vegetative stages, with no significant correlation at the reproductive stage. These findings suggest that selecting for higher early biomass accumulation under saline conditions could improve salinity tolerance, as early shoot vigour is related to higher reproductive success. However, since reproductive-stage biomass did not correlate with yield, breeding efforts should prioritize traits that maintain assimilate allocation to pods under salinity stress.

In terms of molecular insights, the GO enrichment analysis revealed that genes derived from many functional categories were highly enriched at the early vegetative and reproductive stages (Fig. 6), with no significant functional categories identified at the late vegetative stage. Genes involved in abiotic stress responses, organic substance metabolism, cellular processes, and plasma membrane integrity were recurrently enriched at both the vegetative and reproductive stages, suggesting their involvement in salinity stress adaptation across growth stages. At the early vegetative stage, the strong enrichment of genes related to lipid-steroid metabolism, signal transduction, oxidative stress and negative growth regulators suggests a rapid osmotic stress response, consistent with the first phase of salinity stress (Munns 1993; Park et al. 2016). Specifically, genes associated with lipid-steroid metabolism may regulate membrane fluidity to mitigate osmotic imbalance (Dutta 2023; Pan et al. 2023), while ROS-related pathways could indicate activation of oxidative stress responses (Hasanuzzaman et al. 2021; Athar et al. 2022). The enrichment of genes related to negative growth regulation suggests that mungbean prioritizes stress mitigation over biomass accumulation during early-stage stress exposure (Zhou et al. 2024). During the reproductive stage, late-response genes involved in transcriptional and posttranscriptional regulation, metabolic adjustments, and redox homeostasis likely facilitate long-term adaptation to ionic stress (Park et al. 2016; Zhao et al. 2020). The upregulation of transcription factors and biosynthetic pathways likely facilitates stress adaptation through hormonal signalling and metabolic remodelling (van Zelm et al. 2020; Shiade et al. 2024). Additionally, the enrichment of genes involved in anatomical structure development may indicate changes in reproductive organ formation to compensate for stress-induced damage (Yao et al. 2023). This delayed stress response is consistent with the observed phenotypic strategy to balance resource allocation between stress defence and reproductive success under salinity conditions (Munns et al. 2020). Future studies should integrate physiological measurements such as stomatal conductance and Na⁺/Cl⁻ accumulation to directly relate salinity stress traits with molecular responses. Transcriptomic or proteomic analyses at different growth stages could further elucidate stage-specific regulatory pathways controlling osmotic and ionic stress tolerance.

## Conclusion

In conclusion, the mungbean mini-core germplasm collection exhibited a diverse range of phenotypic responses to salinity stress at the early vegetative, late vegetative and reproductive stages, indicating significant diversity within mungbean that could be harnessed in breeding programs once the salinity tolerance is validated in the field. The apparent discrepancy in salinity tolerance observed across the three growth stages and the identification of multiple genomic regions and candidate genes suggest that salinity tolerance is a complex trait with distinct mechanisms involved at different stages of plant development. The findings emphasize the importance of stage-specific breeding approaches and highlight candidate genes with potential applications in enhancing salinity tolerance in mungbean. The SNP dataset derived from low-density DArTseq genotyping was as equally capable and effective as the high-density WGRS dataset for GWAS and GP analyses. This outcome highlights that genotyping can be performed in a cost-effective manner for practical commercial breeding applications (e.g. genomic selection) without compromising on power and genetic resolution by using reduced representation genotyping-by-sequencing methods like DArTseq. After validation, the identified markers will be useful for marker-assisted selection in breeding salt-tolerant mungbean varieties. Moreover, the salt-tolerant genotypes identified in this study could be valuable genetic resources for developing salt-tolerant mungbean varieties with the potential to increase yield and productivity in salt-affected areas. Given the genetic basis of mungbean salinity tolerance and the potential for marker efficiencies, further work is needed to validate the results by evaluating a larger number of genotypes with different genetic backgrounds across multiple years and locations under saline field conditions.

## Supplementary Information

Below is the link to the electronic supplementary material.

Supplementary file1 (DOCX 875 KB)

Supplementary file2 (XLSX 356 KB)

## Data Availability

The datasets generated during and/or analysed during the current study are available from the corresponding author on reasonable request.
