# Supplementary material for: Dissecting the genetic and phenotypic basis of salinity tolerance in mungbean: insights from multi-stage phenotyping, GWAS and genomic prediction
Source: Theor Appl Genet. 2025 Aug 9;138(9):207. doi: 10.1007/s00122-025-04983-z (PMC12335406; doi:10.1007/s00122-025-04983-z)
Supplement: Supplementary file 1 — Supplementary file1 (DOCX 875 KB) [file 122_2025_4983_MOESM1_ESM.docx]

**Dissecting the Genetic and Phenotypic Basis of Salinity Tolerance in Mungbean: Insights from Multi-Stage Phenotyping, GWAS and Genomic Prediction**

**Md Shahin Iqbal ^1,2,3^*****, Candy M Taylor ^4^, Lukasz Kotula ^2^, Al Imran Malik ^1,2,5^ and**

**William Erskine ^1,2^**

^1^ Centre for Plant Genetics and Breeding, The UWA School of Agriculture and Environment, The University of Western Australia, Perth, WA 6009, Australia

^2^ The UWA Institute of Agriculture, The University of Western Australia, Perth, WA 6009, Australia

^3^ Pulses Research Center, Bangladesh Agricultural Research Institute, Ishurdi, Bangladesh

^4^ The Commonwealth Scientific and Industrial Research Organisation (CSIRO), Agriculture and Food, Floreat, WA 6010, Australia

^5^ International Center for Tropical Agriculture (CIAT-Asia), Lao People’s Democratic Republic Office, Vientiane, Laos

*Corresponding author: Md Shahin Iqbal; E-mail address: [mdshahin.iqbal@research.uwa.edu.au](about:blank); shahinsarvi@gmail.com

**Supplementary figures**


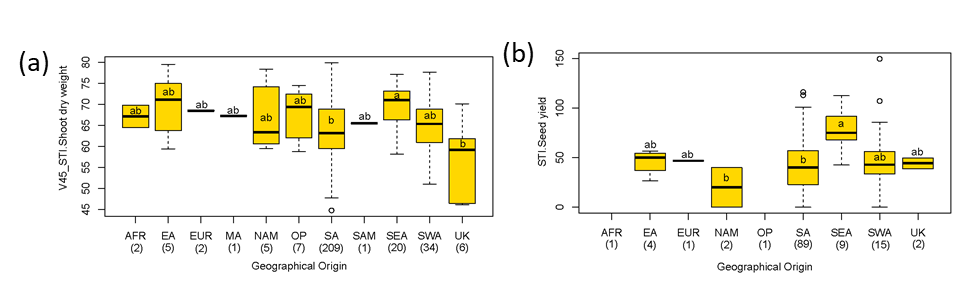


**Fig. S1** Association between V45_STI.Shoot dry weight (a) and STI.Seed yield (b) with geographical region of origin. Box plot represents means of each trait (mid-line of box plot), standard error (box plot length), and minimum and maximum values (whisker bars). Numbers in brackets denote the number of genotypes in a region. AFR: Africa; EA: East Asia; EUR: Europe; MA: Central America; NAM: North America; OP: Oceania and the Pacific; SA: South Asia; SAM: South America; SEA: South East Asia; SWA: South West Asia; UK: unknown.


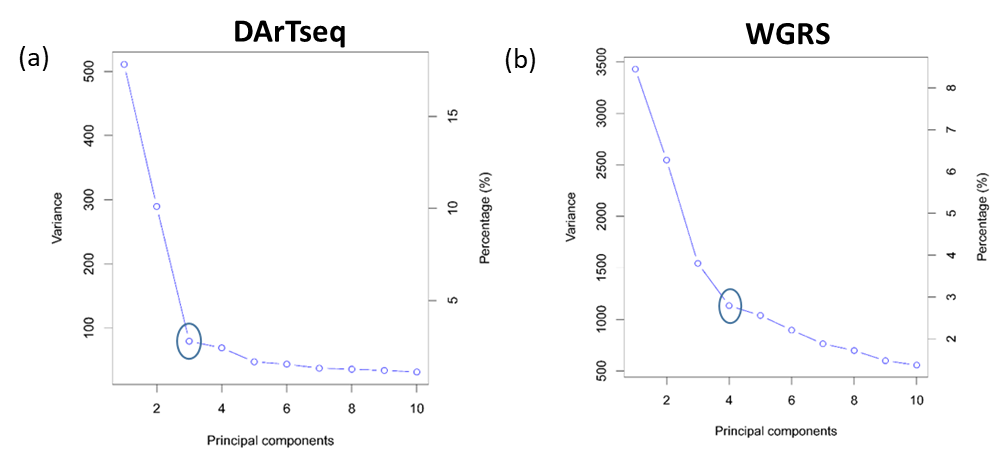


**Fig. S2** Scree Plot of PCA analysis (the elbow point) suggest 3 subpopulations in DArTseq SNPs (a) and 4 subpopulations in WGRS SNPs (b). Ellipse indicates the elbow point.


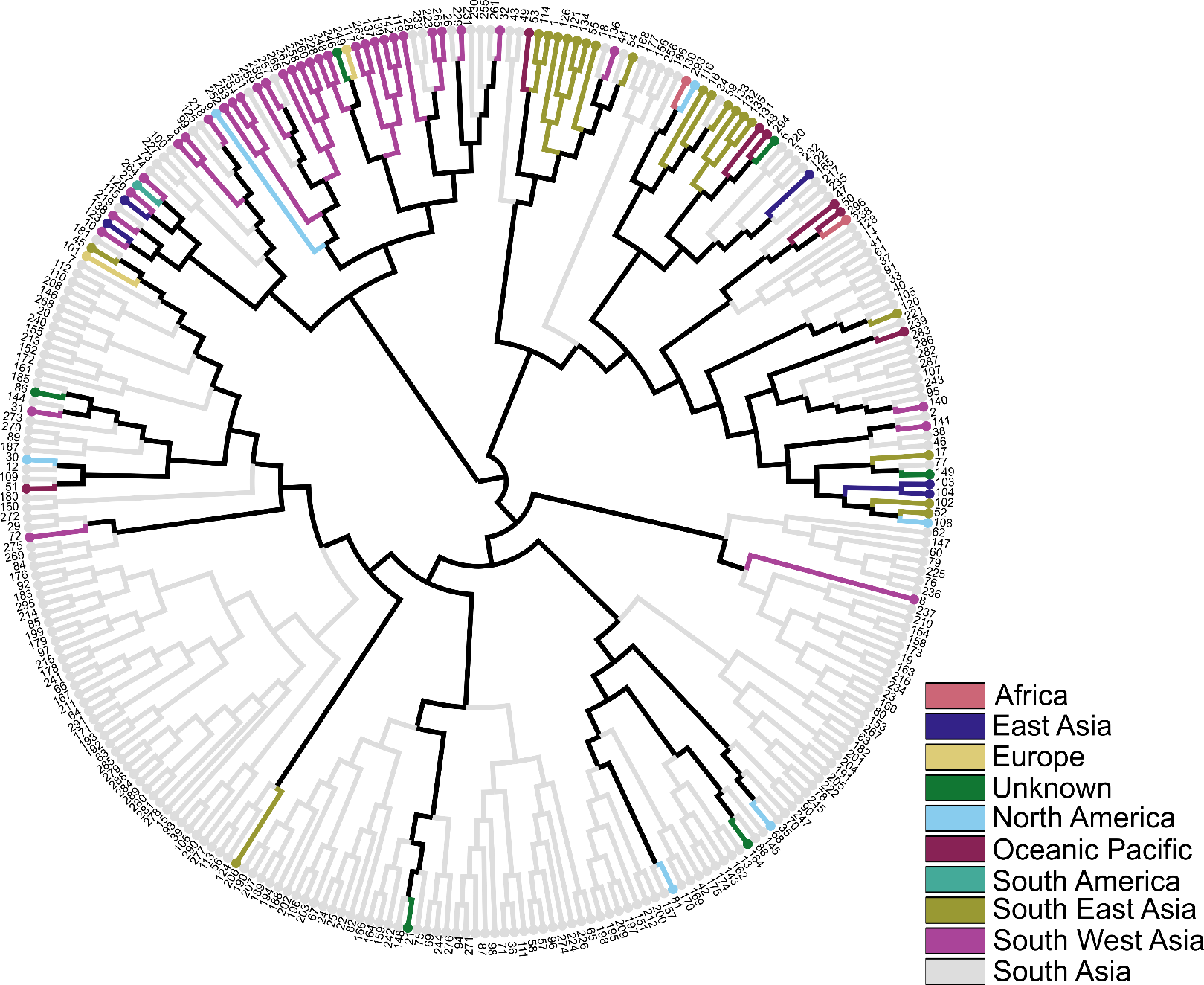


**Fig. S3** Neighbour-joining tree among the 292 mungbean mini-core genotypes of WGRS SNPs


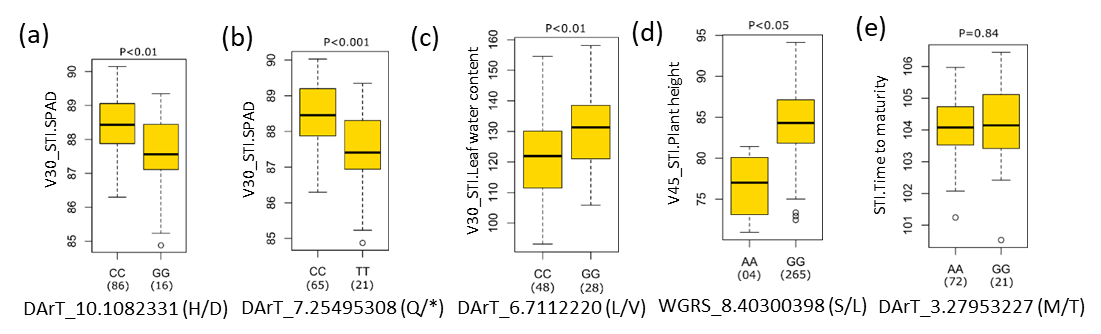


**Fig. S4** Boxplot for the five non-synonymous (missense or nonsense) SNPs associated with V30_STI.SPAD (a, b), V30_STI.Leaf water content (c), V45_STI.Plant height (d), and STI.Time to maturity (e). Genotypes were divided into two groups at each locus based on the allele type. Significant differences between the phenotypes of these two allele groups were analyzed by t-test (P < 0.05). The number of genotypes harboring the corresponding allele is shown in brackets at the bottom.


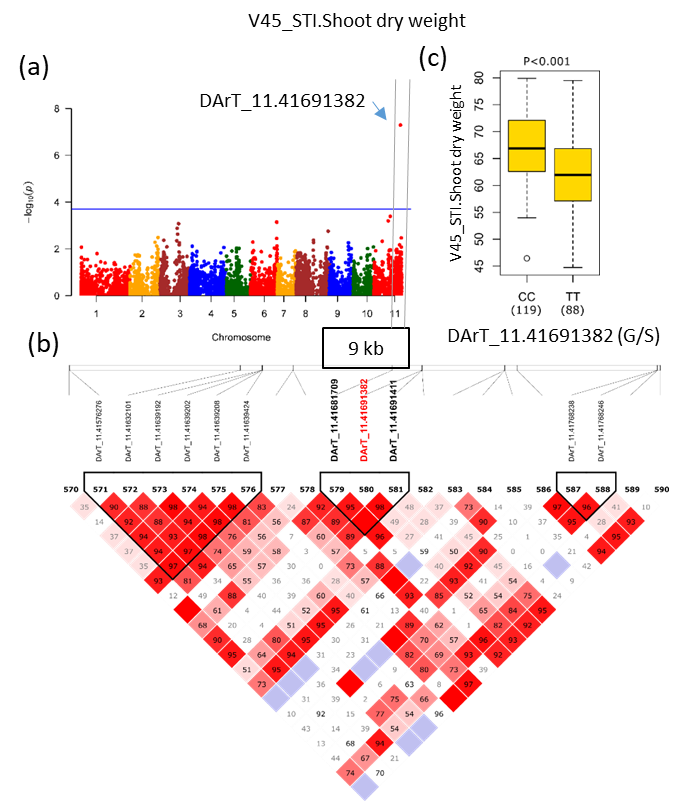


**Fig. S5** Manhattan plot showing significant marker-trait association of V45_STI.Shoot dry weight (a). Significant functional markers are highlighted with arrow. The horizontal blue line represents the significance threshold (P-value <1.89 × 10^−4^ ). LD heatmap (b) surrounding the significant SNP DArT_11.41691382 on chromosome 11 showing 3 SNPs in the candidate region for V45_STI. Shoot dry weight. Each coloured diamond shows correlation between the two markers. Boxplot for the missense SNP DArT_11.41691382 (c). Genotypes were divided into two groups at each locus based on the allele type. Significant differences between the V45_STI.Shoot dry weight of these two allele group was analysed using t-test (P<0.05). The number of genotypes harbouring the corresponding allele is shown in brackets at the bottom.
